# Emerging entities: high-grade/large B-cell lymphoma with 11q aberration, large B-cell lymphoma with *IRF4* rearrangement, and new molecular subgroups in large B-cell lymphomas. A report of the 2022 EA4HP/SH lymphoma workshop

**DOI:** 10.1007/s00428-023-03590-x

**Published:** 2023-08-09

**Authors:** Leticia Quintanilla-Martinez, Camille Laurent, Lorinda Soma, Siok-Bian Ng, Fina Climent, Sarah L. Ondrejka, Alberto Zamo, Andrew Wotherspoon, Laurence de Leval, Stefan Dirnhofer, Lorenzo Leoncini

**Affiliations:** 1 Institute of Pathology and Neuropathology, Eberhard-Karls-University of Tübingen and Comprehensive Cancer Center, University Hospital Tübingen, Liebermeisterstrasse 8, 72076 Tübingen, Germany; 2 Cluster of Excellence iFIT (EXC2180) “Image-guided and functionally Instructed Tumor therapies” Eberhard-Karls-University, Tübingen, Germany; 3 Department of Pathology, Toulouse University Hospital Center, Cancer Institute, University of Toulouse-Oncopole, Toulouse, France; 4 Department of Pathology, City of Hope National Medical Center, Duarte, CA USA; 5 Department of Pathology, Yong Loo Lin School of Medicine, National University of Singapore, Singapore, Singapore; 6 Cancer Science Institute of Singapore, National University of Singapore, Singapore, Singapore; 7 Department of Pathology, Hospital Universitari de Bellvitge-IDIBELL, L’Hospitalet de Llobregat, Barcelona, Spain; 8 Pathology and Laboratory Medicine Institute, Cleveland Clinic, Cleveland, OH USA; 9 Institute of Pathology, University of Würzburg, Würzburg, Germany; 10 Department of Histopathology, Royal Marsden Hospital, London, UK; 11 Institute of Pathology, Department of Laboratory Medicine and Pathology, Lausanne University Hospital and Lausanne University, Lausanne, Switzerland; 12 Institute of Medical Genetics and Pathology, University Hospital Basel, University of Basel, Basel, Switzerland; 13 Department of Medical Biotechnology, Section of Pathology, University of Siena, Siena, Italy

**Keywords:** High-grade/large B-cell lymphoma, 11q aberration, *IRF4*-rearrangement, Plasmablastic transformation, *CCND1*-R in DLBCL, B-ALL with *MYC*-R, Primary bone lymphoma

## Abstract

Emerging entities and molecular subgroups in large B-cell lymphomas (LBCLs) were discussed during the 2022 European Association for Haematopathology/Society for Hematopathology workshop in Florence, Italy. This session focused on newly recognized diseases and their diagnostic challenges. High-grade/large B-cell lymphoma with 11q aberration (HG/LBCL-11q) is defined by chromosome 11q-gains and telomeric loss. FISH analysis is recommended for the diagnosis. HG/LBCL-11q can occur in the setting of immunodeficiency, including ataxia-telangiectasia, and predominates in children. The morphological spectrum of these cases is broader than previously thought with often Burkitt-like morphology and coarse apoptotic bodies. It has a Burkitt-like immunophenotype (CD10+, BCL6+, BCL2−) but MYC expression is weak or negative, lacks *MYC* rearrangement, and is in contrast to Burkitt lymphoma 50% of the cases express LMO2. LBCL with *IRF4* rearrangement (LBCL-*IRF4*) occurs mainly in the pediatric population but also in adults. LBCL-*IRF4* has an excellent prognosis, with distinguishing molecular findings. *IRF4* rearrangements, although characteristic of this entity, are not specific and can be found in association with other chromosomal translocations in other large B-cell lymphomas. Other molecular subgroups discussed included primary bone diffuse large B-cell lymphoma (PB-DLBCL), which has distinctive clinical presentation and molecular findings, and B-acute lymphoblastic leukemia (B-ALL) with *IGH::MYC* translocation recently segregated from Burkitt lymphoma with TdT expression. This latter disorder has molecular features of precursor B-cells, often tetrasomy 1q and recurrent *NRAS* and *KRAS* mutations. In this report, novel findings, recommendations for diagnosis, open questions, and diagnostic challenges raised by the cases submitted to the workshop will be discussed.

**Supplementary Information:**

The online version contains supplementary material available at 10.1007/s00428-023-03590-x.

## Introduction

Diffuse large B-cell lymphoma (DLBCL) accounts for ~40% of non-Hodgkin lymphomas (NHL), comprising specific subtypes or disease entities [1–3]; however, most cases fall into the category of “not otherwise specified” (NOS). DLBCL, NOS represents a heterogeneous group that has been divided based on the gene expression profile (GEP) of B-cells or cell of origin (COO) into two subtypes: germinal center B-cell (GCB), and activated B-cell (ABC) [4]. A small subset of DLBCL is considered “unclassified” and not fitting in any category. Nonetheless, this COO binary classification is insufficient to capture the heterogeneity and the complexity of this disease. The characterization of *MYC* rearrangement (R) associated with *BCL2*-R and/or *BCL6-R* as well as double-hit/dark zone signature has further emphasized the biological diversity of DLBCL [5–7]. More recently, the integration of molecular data including structural variants, mutational profile, and somatic copy-number alterations has identified at least 7 DLBCL subgroups with putative clinical relevance, demonstrating the complexity of DLBCL [8–11]. Among other LBCL with specific sites of involvement and characteristic pathological features are primary mediastinal B-cell lymphoma, and the group of LBCL of the central nervous system, vitreoretinal compartment, and testes, considered immune privileged sites [12, 13]. Others arising in the setting of immune dysfunction or in association with EBV or HHV8 infectious agents have been also identified as specific disease entities [14–16].

The presence of specific and recurrent genetic alterations has identified new molecular subgroups of LBCL. Among them, LBCL with *IRF4* rearrangement (LBCL-*IRF4*) [17–19], and high-grade/large B-cell lymphoma with 11q aberration (HG/LBCL-11q) with features resembling Burkitt lymphoma (BL) without *MYC*-R, emerged as new entities in the 2022 International Consensus Classification (ICC) and in the 5th edition of the WHO classification [20–22]. However, due to their rarity, some questions regarding their distinct pathological and molecular features or their clinical presentation and outcome need to be addressed. In addition, the issue of what is the best strategy to diagnose these new entities and what technique would be most appropriate in routine diagnosis must be addressed. Other peculiar and rare LBCL such as primary bone diffuse LBCL (PB-DLBCL) or those harboring infrequent and/or complex cytogenetic abnormalities are sometimes difficult to classify accurately.

The major theme of the 2022 European association for Haematopathology (EA4HP) and Society for Hematopathology (SH) workshop in Florence, Italy, was “Provisional and emerging disease entities.” A session was dedicated to emerging entities and molecular subgroups in LBCL. Fifty-six cases were submitted, representing the many challenges in the diagnosis of these new recognized lymphomas both in children and adults. The cases were divided into the following thematic groups to illustrate diagnostic dilemmas and/or interesting biological features:

1. High-grade/large B-cell lymphoma with 11q aberration (HG/LBCL-11q)
2. Large B-cell lymphoma with *IRF4* rearrangement (LBCL-*IRF4*)
  - In pediatric population and young adults (<25 years)
  - In adult patients (>25 years)
3. Aggressive B-cell lymphomas with *IRF4* and *BCL2/MYC/CCND1* rearrangements
4. Other molecular groups in large B-cell lymphomas

### High-grade/large B-cell lymphoma with 11q aberrations

HG/LBCL-11q is an aggressive mature B-cell lymphoma with a characteristic chromosome 11q-gain/loss pattern, displaying variable morphological features ranging from a typical BL-like morphology to a more intermediate or blastoid appearance [2, 20, 23]. HG/LBCL-11q cases occurs as a localized nodal (most commonly head and neck; 60–70%) or extranodal disease (gastro-intestinal tract 30–40%). The defining genetic event is a complex aberration involving the long arm of chromosome 11 (11q), showing a gain in 11q23.2-23.3 and a telomeric loss in 11q24.1-qter, in the absence of a *MYC*-R. Rare cases do not have the gain of 11q23.3 [21, 22, 24]. The 5th WHO lymphoma classification and the 2022 ICC recognize cases with only telomeric loss and/or solely telomeric loss of heterozygosity as *bona-fide* cases of HG/LBCL-11q [25, 26]. However, further studies are needed to corroborate whether these cases belong to the same disease. The presence of 11q23.3 gains, as the only alteration, is considered non-specific, and therefore not enough for the diagnosis. In contrast to BL, mutations in the ID3-TCF3 pathway, recognized as the biological hallmark of BL, are not detected in HG/LBCL-11q. The mutational landscape of HG/LBCL-11q is closer to that of DLBCL of GCB-type, and *GNA13* mutations are observed in about 50% of cases [21, 22]. These new data, together with the absence of the *IGH::MYC* rearrangement, suggest that HG/LBCL-11q represents a different mature aggressive B-cell lymphoma [20].

In the workshop, 20 cases were submitted with the diagnosis of HG/LBCL-11q (Supplemental Table 1). The diagnosis was confirmed in 18 cases that were considered to represent good examples of HG/LBCL-11q. The clinicopathological features are summarized in Table 1. Male patients predominated (M/F=13/5) with median age at presentation of 22.5 years (range 7–72 years). Thirteen cases were children and young adults (range 7–31 years), whereas 5 patients were >40 years (44–72 years). From the cases submitted, 10 cases showed nodal presentation, whereas 8 cases involved extranodal sites. One patient (LYWS-1073, submitted by Rex Au-Yeng) showed various sites of involvement at different time points. The disease was first diagnosed in a cervical lymph node, and at relapse 2 years later presented in the tonsil. Interestingly, 2 cases occurred in the setting of primary immunodeficiency (LYWS-1125 presented by Olga Balague and LYWS-1442 submitted by Peggy Dartigues). Both cases were known to have ataxia-telangiectasia (A-T) diagnosed at the age of 11 and 13 years, respectively. A-T is the most common DNA repair disorder, caused by the presence of biallelic pathogenic variants in the *ATM* gene, and characterized by a very high risk of developing hematological malignancies during childhood, especially DLBCL and T-cell acute lymphoblastic leukemia (ALL), but rarely BL [27]. Yet, the association with HG/LBCL-11q has not been reported. This peculiar 11q-gain/loss aberration has been described to be particularly frequent in posttransplant patients, who develop B-cell lymphomas with Burkitt-like morphology [28]. It has also been described in the setting of HIV infection [29–31].

**Table 1:** Summary of the clinical, morphological, and genetic features of 18 cases with the diagnosis of high-grade/large B-cell lymphoma with 11q aberration

Clinicopathological features	High-grade B-cell lymphoma with 11q aberration/large B-cell lymphoma with 11q aberration
Age	Median 22.5 years (range 7–72 years)
Sex	M:F 2.5:1
Localization	
-Tonsils	4 cases (22%)
-Lymph nodes	7 cases (38%)
-Abdominal mass	5 cases (28%)
-Ovary	1 case (6%)
-Not available	1 case (6%)
Clinical stage	
- Stage 1/2	12 cases (67%)
- Stage 3/4	6 cases (33%)
Cytology	
-Blastoid	4 cases (22%)
-Intermediate, Burkitt like	9 cases (50%)
-Large	1 case (6%)
-Not available	4 cases (22%)
Immunophenotype	
-CD10	17 cases (94%)
-BCL6	17 cases (94%)
-LMO2*	7 cases (38%)
-BCL2	1 case (6%)
-MYC**	18 cases (100%)
-MUM1	2 cases (12%)
FISH	
-*11q* gain	18 cases (100%)
-*11q* loss	18 cases (100%)
-*IGH::MYC*	0 (0%)
*-BCL2*	0 (0%)
*-BCL6*	0 (0%)

*Performed only on 15 cases in which the material was available; *M* male, *F* female

**Fig. 1:**
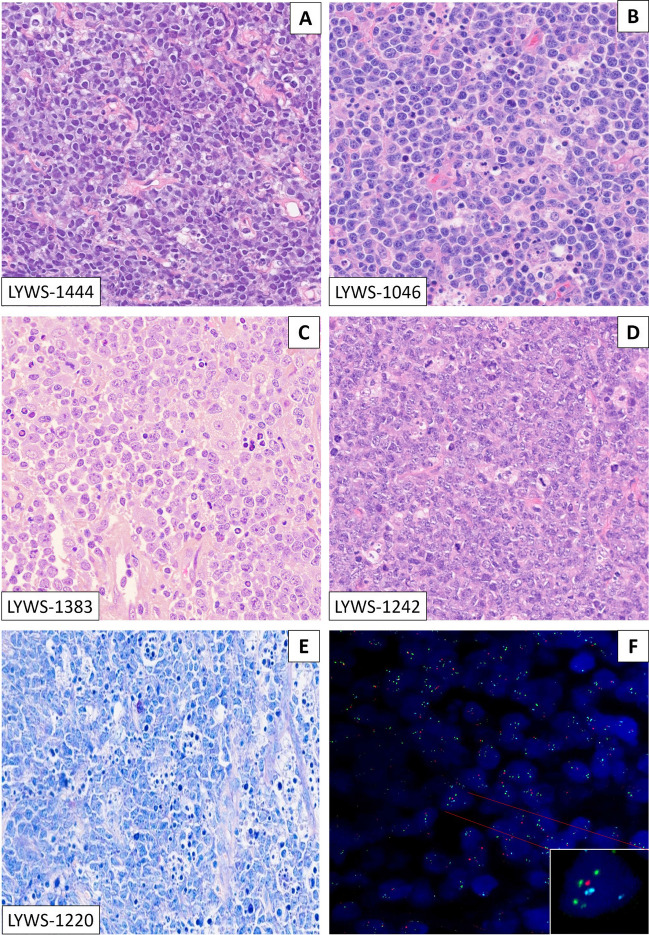
The morphological spectrum of high-grade/large B-cell lymphoma with 11q aberration: the morphological spectrum varied from blastoid (**A**) to cases with intermediate morphology, ranging from cases very similar to BL (**B**) to other more similar to DLBCL (**C**); only one case was submitted with typical large cell morphology (**D**). Starry-sky pattern with conspicuous, coarse apoptotic debris was detected in 5 cases. **E** FISH analysis showed 11q gain/loss in all cases (**F**). **A**–**D** H&E stain; **E** Giemsa stain; **F** FISH analysis with ZytoLight® SPEC 11q gain/loss Triple Color Probe

The morphological spectrum of the submitted cases varied from blastoid to intermediate to large B-cell lymphomas (Fig. 1). The cases with intermediate morphology displayed cellular pleomorphism, variation in nuclear size, shape, and larger nucleoli ranging from cases very similar to BL to other more akin to DLBCL. Only one case (LYWS-1242 submitted by Snjezana Dotlic) showed large cell morphology. In 10 cases, a starry-sky pattern was present, and in 5 cases, coarse apoptotic debris was observed. In a recent study, coarse apoptotic bodies were reported as characteristic of HG/LBCL-11q, and an important morphological feature to suspect the diagnosis and to prompt the FISH analysis [20, 32]. The cases submitted to the workshop confirmed this contention; however, although a helpful feature to suspect the diagnosis, it was not seen in many cases, and therefore, its absence does not exclude the diagnosis. All cases displayed a GCB phenotype applying the Hans algorithm (CD10+, BCL6+), and a high proliferation index (Ki67>90%). However, 3 cases showed MUM1 and BCL2 expression (LYWS-1129; LYWS-1237; LYWS-1217). A recent study demonstrated that 46% of HG/LBCL-11q expresses LMO2, a germinal center marker, using the monoclonal antibody SP51, as opposed to BL, which is negative [21]. Accordingly, 7/15 (46%) cases investigated were LMO2 positive. The workshop cases confirmed that LMO2 is a useful marker to support HG/LBCL-11q over BL in difficult cases. MYC expression varied from case to case, detected in 20 to 60% of tumor cells but always displaying weak staining. In all cases, *MYC-R* was excluded by FISH analysis with break-apart probes (BAP). However, BAP cannot identify all *MYC*-positive cases and 4–20% of cases might remain undetected [33, 34]. The panel tested the cases for *IGH::MYC*, *IGL::MYC*, and *IGK::MYC* translocations. All cases were negative, thus confirming the diagnosis of HG/LBCL-11q. FISH analyses for *BCL2-R* and *BCL6*-R with BAP were negative in all cases.

**Fig. 2:**
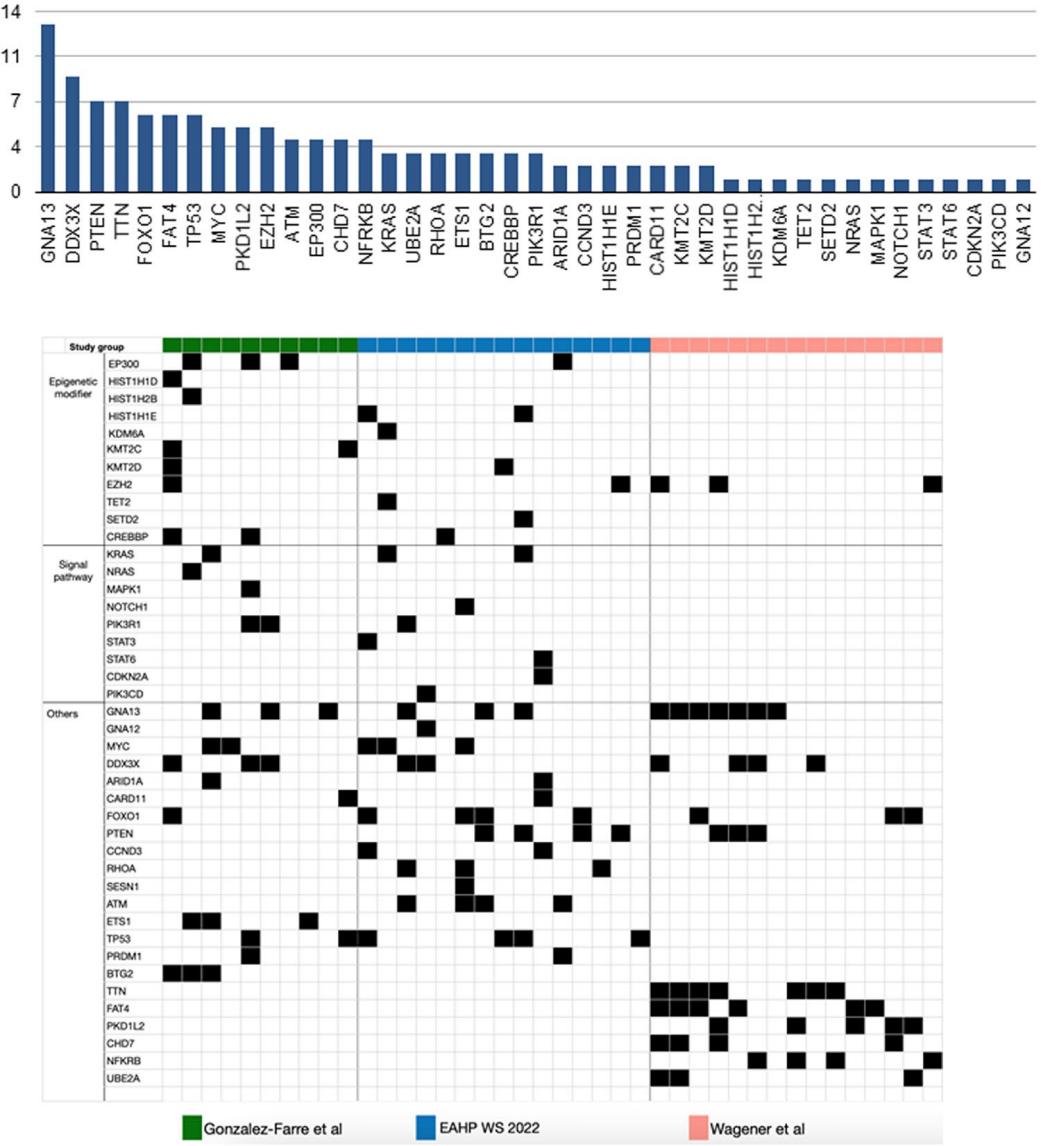
Mutational profile of HG/LBCL-11q cases submitted to the workshop. The mutational landscape of the workshop cases showed similarities but also difference with previous studies. Mutations in *GNA13*, *DDX3X*, *MYC*, *KRAS*, *EZH2*, *CREBBP*, and FOXO1 were confirmed and new mutations in *ATM*, *CCND3*, and *RHOA* were identified. Typical Burkitt lymphoma driver mutations (*ID3* or *TCF3* were not detected). The comparison was performed with the studies of Gonzalez-Farre et al. [21] and Wagener et al. [22]

**Fig. 3:**
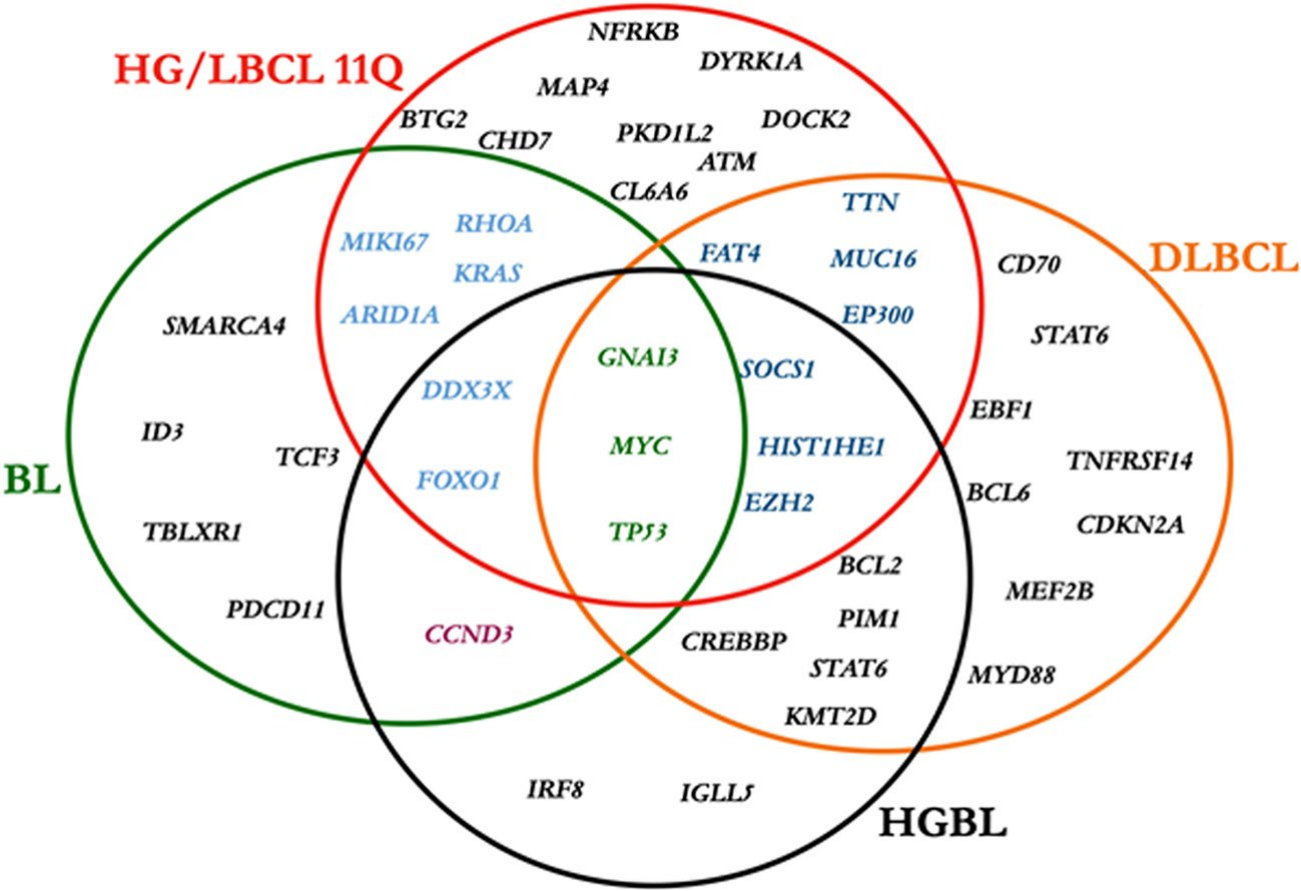
Comparison of the mutational patterns of Burkitt lymphoma (BL), high-grade/large B-cell lymphoma (HG/LBCL)-11q, high-grade B-cell lymphoma (HGBL) wit double/triple hit, and diffuse large B-cell lymphoma (DLBCL). Mutations in *GNA13*, *MYC*, and *TP53* are shared by BL, HG/LBCL-11q, HGBL, and DLBCL. *BTG2* and *DYRK1A* gene mutations are considered typical for HG/LBCL-11q

In 5 cases, the results of next-generation sequencing (NGS) were reported by the submitters. In the remaining cases, for which material was available, the panel performed NGS analysis with a custom panel of 80 genes. The results are reported in Fig. 2 and Supplemental Table 2. The mutational landscape of the workshop cases showed similarities but also differences with previous studies [21, 22]. The previously reported mutations in *DDX3X*, *MYC*, *KRAS*, *EZH2*, *CREBBP*, and *FOXO1* were confirmed. Typical BL driver mutations (*ID3* or *TCF3*) were not identified; however, there were some differences for genes reported to be more frequently mutated in HG/LBCL-11q. In the workshop cases, mutations in *PTEN*, *TP53*, *FOXO1*, and *RHOA* were frequently demonstrated. Of note, mutations in *ATM*, *CCND3*, and *RHOA* have not been previously described in HG/LBCL-11q. Of interest, in addition to the two cases with A-T (LYWS-1125 and LYWS-1442), *ATM* mutations were found in two additional cases (LYWS-1217 and LYWS-1220), with an allele frequency of 49% and 39%, respectively. These mutations may well represent heterozygous germline variants, which have been associated also with increased risk for neoplasia [35, 36]. Thus, these findings further support and expand the association of HG/LBCL-11q with immunodeficiency syndromes. Finally, Fig. 3 shows a comparison of the mutational patterns of BL, HG/LBCL-11q, high-grade B-cell lymphoma (HGBL) with double/triple-hit, and DLBCL. Mutations in *GNA13*, *MYC*, and *TP53* are shared by BL, HG/LBCL-11q, HGBL, and DLBCL. *BTG2* and *DYRK1A* gene mutations are considered typical for HG/LBCL-11q, engaged in FOXO1 and cyclin regulation, and in the TP53 pathway, respectively.

Information regarding the outcome was available in 15 cases. Complete remission (CR) was achieved in 11 cases, 8 pediatric, and 3 adult patients; however, treatment was variable (R-CHOP, DA-R-EPOCH, or R-EPOCH). Three cases relapsed, and only one case died of lymphoma after 2 weeks from the diagnosis. Interestingly, all cases in the pediatric group received BL protocols achieving CR, while the three patients that relapsed were adults (LYWS-1224; LYWS-1234; LYWS-1444), received variable treatments, and carried *TP53* mutations.*TP53* mutations have not been reported in HG/LBCL-11q, so far; nonetheless, it is remarkable that all cases were adults and had a bad prognosis. The question is whether *TP53* mutation is a marker of clonal progression or whether cases with *TP53* mutations and 11q aberrations in adults are more closely related to DLBCL, NOS. Additional mutations in these cases included *HIST1HE1*, *KMT2D*, *SETD2*, *PTEN NSD2*, and *STAT3*, which are more characteristic of DLBCL. However, further studies are warranted to answer this question.

In two cases (LYWS-1171 submitted by Fang Liu and LYWS-1237 submitted by Julie Bruneau), the panel could not confirm the diagnosis of HG/LBCL-11q. In case LYWS-1171, the FISH analysis showed only gain of 11q23.3 but not loss at 11q24.1 region. As discussed above, the 11q gain is not specific for HG/LBCL-11q because it also occurs in other mature aggressive B-cell lymphomas. In this case, because of lack of material, the panel could not perform additional studies and rendered the final diagnosis of HGBL, NOS. The second case LYWS-1237, with the proposed diagnosis of “BL with unusual BCL2 expression,” showed morphology that was compatible with BL but the strong BCL2 and MUM1 expression, along with a complex karyotype, *MYC*-R and 11q aberration, were quite atypical for a BL diagnosis. In contrast to HG/LBCL with 11q aberration, this case showed strong, homogeneous MYC expression. The NGS analysis confirmed a mutational profile more compatible with HGBL, NOS. The recommendation of the panel was to classify these cases (*MYC*-R with 11q aberration) as HGBL, NOS, based on the mutational analysis, although further studies are necessary to answer this question.

Important issues were raised during the discussion of the session, in particular the best technical approach to confirm the diagnosis. The recommendation of the panel was to perform FISH analysis for 11q aberration with commercially available probes in all *MYC*-R negative cases with an intermediate/blastoid or Burkitt-like morphology. However, in cases where the telomeric loss cannot be demonstrated by FISH, it is necessary to perform additional molecular analysis (Oncoscan/CGH) to confirm or exclude the diagnosis. Another key issue discussed was if FISH analysis for 11q should be performed in all *MYC*-R BL, particularly in those cases with an atypical phenotype, to identify potential 11q aberrations with *MYC*-R, as demonstrated in case LYWS-1237. Currently, it is not recommended to do FISH for 11q in typical BL cases with *MYC*-R; however, this should be encouraged for cases with *MYC*-R and atypical BL morphology or phenotype.

### Large B-cell lymphoma with *IRF4* rearrangement

LBCL-*IRF4* was introduced, as a provisional entity, in the 2016 revised 4th WHO classification within the group of follicular lymphomas (FLs) because of its frequent follicular growth pattern and excellent prognosis [2, 37], and now is recognized as a definite entity, both in the 2022 International Consensus Classification (ICC), where it remains in the group of FL [25, 38], and in the 5th WHO classification, where it was moved to the group of LBCL [26]. LBCL-*IRF4* occurs mainly in children and young adults with distinctive clinical presentation preferentially involving the tonsils and mucosa-associated lymphoid tissue comprising Waldeyer’s ring, head and neck lymph nodes, and less frequently the intestine. It has a slight male predilection and presents as a localized disease (stages I–II) with excellent prognosis, regardless of the large cell cytology and the growth pattern [17, 19, 24]. It has been estimated that in the pediatric population, around 20% of morphologically FL (grade 3) and DLBCL harbor an *IRF4* chromosomal translocation but represent only 1–2% of all lymphomas in children and adolescents [24]. Morphologically, LBCL-*IRF4* is often follicular and diffuse but it might be purely follicular or diffuse. The tumor cells are medium or large in size and show the morphology of centroblasts or blastoid cells sometimes with a starry-sky pattern and high proliferation rate. The tumor cells express B-cell markers and often germinal center markers including CD10 and BCL6 together with the constitutive expression of MUM1/IRF4. *IGH::IRF4* rearrangements are detected in most cases. LBCL-*IRF4* lacks *BCL2-R* and *MYC-R*; however, many cases carry *BCL6*-R (35%) [17]. Molecular studies have revealed a GCB-type GEP, despite the constitutive expression of IRF4, and frequent mutations in *IRF4* and NF-kB-related genes (*CARD11*, *CD79B*, *MYD88*) [39]. The most frequent chromosomal alteration is loss of 17p13, where *TP53* gene is located [39, 40].

It has been shown that biology and pathogenesis in DLBCL is age-dependent and the cutoff of 18 years used in clinical practice seems rather arbitrary and does not reflect DLBCL biology [41, 42]. Molecular features suggest that a better cutoff might be in the mid-thirties [41]. Accordingly, two recent studies have shown that LBCL-*IRF4* in young adults (<40 years) share the localization, morphology and genetics of pediatric patients [40, 43]. In the workshop, 16 cases were submitted with the diagnosis of LBCL-*IRF4*; for practical purposes, an arbitrary cutoff of 25 years was set to separate pediatric and young adults from adult patients to compare the clinico-pathological features of the two groups. All cases were considered to represent good examples of LBCL-*IRF4*, some with unique features.

#### LBCL-IRF4 in patients < 25 years

**Fig. 4:**
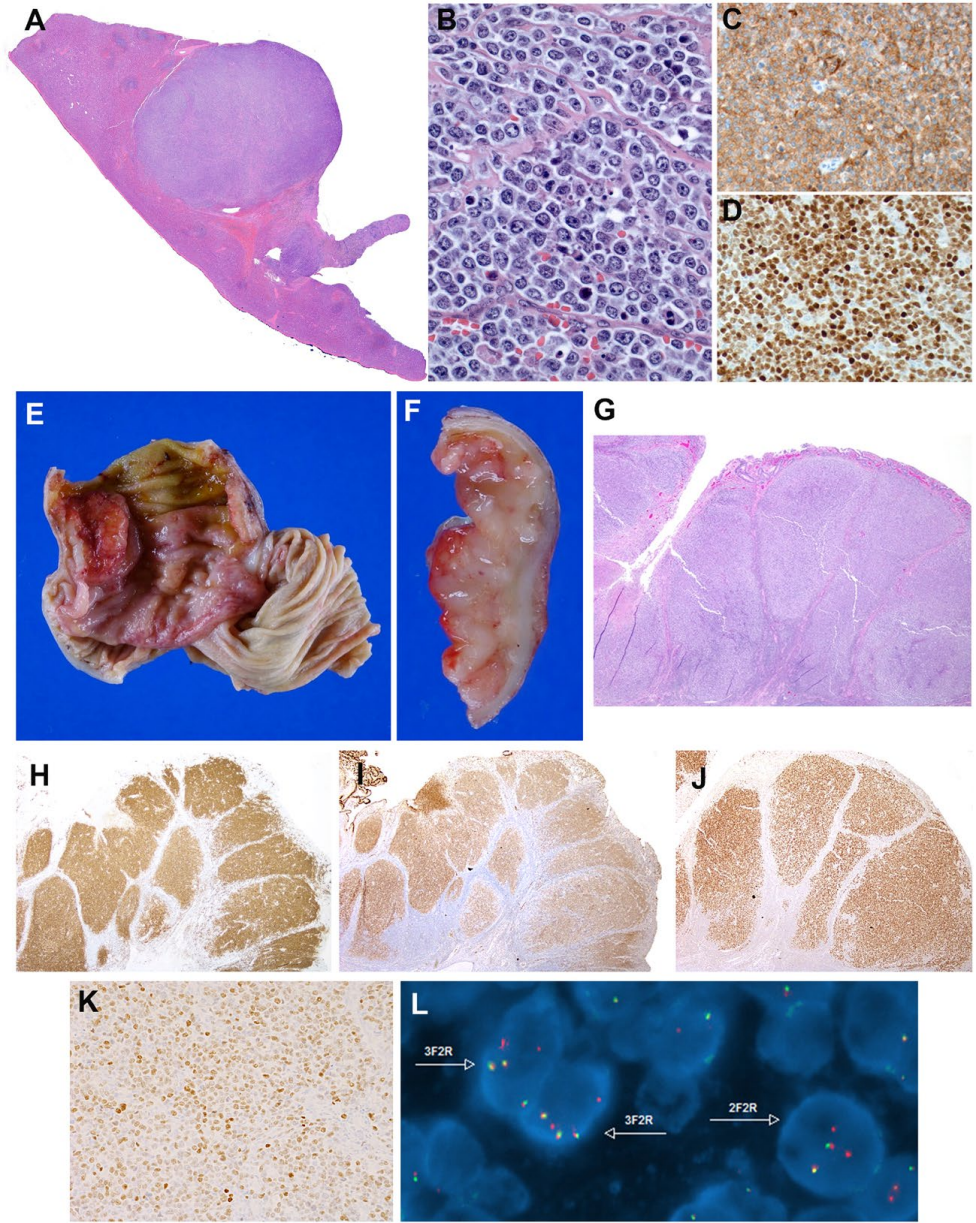
Histologic and immunophenotypic features of large B-cell lymphoma with *IRF4*-rearrangement presenting in extranodal sites. **A**–**D** Case LYWS-1163 courtesy of E. Shuyu. **A** Spleen section showing a well-circumscribed nodular infiltration. **B** The tumor is composed of large centroblastic lymphoid cells with open chromatin, several large nuclei, and abundant cytoplasm. **C** The tumor cells are CD10 and **D** IRF4/MUM1 strongly positive. **E**–**L** Case LYWS-1112 courtesy of D. Wang. **E** Terminal ileum with a 2.2-cm large polypoid mass. **F** The intestinal crossed section shows a white soft mass infiltrating the mucosa, submucosa, and the muscularis propria. **G** H&E section reveals a follicular lymphoid infiltrate with large, back-to-back follicles. The follicles are composed of medium to large-sized centroblasts. **H** The tumor cells are positive for CD79a, **I** CD10, **J** BCL6, and **K** IRF4/MUM1. **L** Interphase FISH analysis using break apart probes for *IRF4*. Most cells have 3 fusion signals (yellow) and 2 red signals with loss of the green signals indicating an *IRF4* translocation

**Fig. 5:**
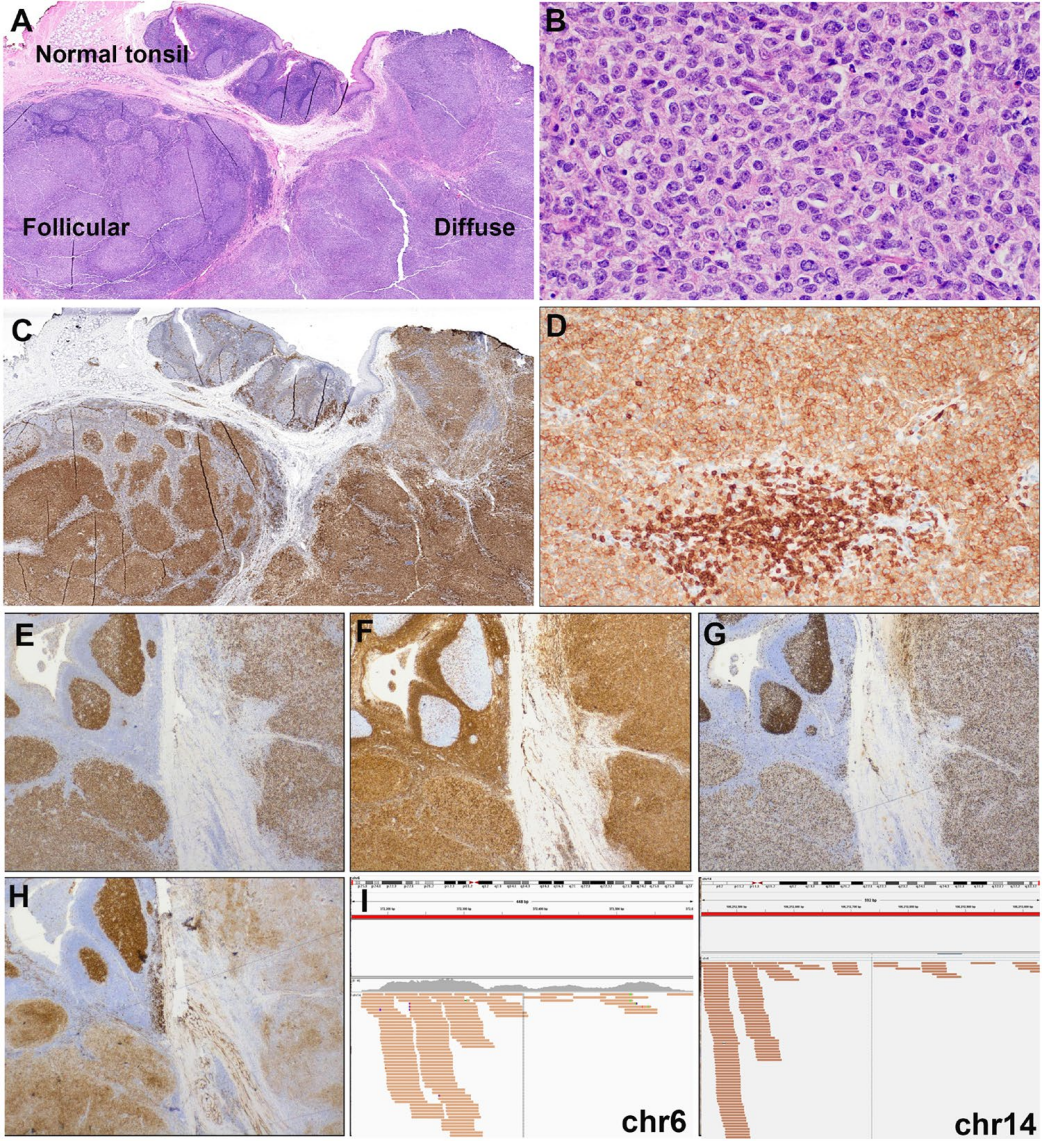
Histologic and immunophenotypic features of large B-cell lymphoma with *IRF4*-rearrangement presenting in the tonsil. Case LYWS-1279 courtesy of R. Mariani. **A** Panoramic view of a tonsil showing residual normal tonsil areas and lymphoma infiltration with follicular and diffuse pattern. **B** Higher magnification shows a lymphoid infiltrate composed of large-sized centroblasts. **C** IRF4/MUM1 stain shows in the normal residual lymphoid tissue few positive plasma cells. The left side shows a follicular growth pattern whereas the left side reveals a diffuse growth pattern. **D** The tumor cells show an aberrant CD5 expression. Note the strong CD5 expression of the reactive T cells. **E** The tumor cells are positive for BCL6, and **F** BCL2. **G** The MIB1 stain shows the normal polarization of residual germinal centers, whereas the tumor shows a proliferation of approximately 80% in both the follicular and in the diffuse areas. **H** The CD10 stain is strong and homogeneous positive in the residual germinal center whereas the stain is weak in the follicular areas and partially lost in the diffuse areas. **I** IGV screenshots of chimeric pairs in chromosomes 6 and 14 supporting the presence of *IGH::IRF4* juxtaposition

The cases submitted illustrated the clinical, morphological, and genetic spectrum described in this disease (Table 2 and Supplemental Table 3) [17–19, 24, 44]. There were 5 males and 2 females with a median age of 8 years (range 6–25 years). Four cases presented in the tonsil, and one each in a cervical lymph node, intestine, and spleen. The spleen presentation (Case LYWS-1163, submitted by E. Shuyu) is exceptional and occurred in an 18-year-old male, who presented with a single 10-cm mass (Fig. 4A–D). Importantly all cases were in stage I or II and all the patients were in CR, four after systemic chemotherapy. One case without systemic chemotherapy (Case LYWS-1112, submitted by Dehua Wang) was a 7-year-old boy with a 2.2-cm mass in terminal ileum that was surgically resected due to intussusception (Fig. 4E–L). Morphologically, the lymphoma was follicular with typical phenotype and genotype. This case together with case LYWS-1395 and other cases reported in the literature that achieved CR without systemic treatment raise the question of whether chemotherapy can be reduced or a watch-and-wait strategy can be followed for those cases with follicular growth pattern and localized disease after excision [18, 19, 45]. Accordingly, in the pediatric cohort published by the NHL-Berlin-Frankfurt-Münster group, because of the excellent prognosis of these patients, it was recommended therapy de-escalation in future clinical trials [24]. Morphologically, 3 cases were follicular and diffuse, 2 cases diffuse, and 2 cases follicular. In 6 cases, the immunophenotype revealed co-expression of CD10, BCL6, and MUM1. The abnormal co-expression of these three markers should prompt to investigate the diagnosis of LBCL-*IRF4* [40]. Only one case was CD10 negative (LYWS-1049). As previously reported [17], 3 cases (3/7, 43%) showed weak CD5 expression (LYWS-1279, LYWS-1112, LYWS-1395), and 6 cases (6/7, 86%) expressed BCL2 without t(14;18) translocation. FISH analyses demonstrated in 6/7 cases an *IRF4* break indicating an *IRF4* translocation. In one case, presented by Rachel Mariani (case LYWS-1279), with typical localization, morphology, and immunophenotype (Fig. 5A–I), FISH BAP analysis was negative for *IRF4* break. NGS demonstrated an *IGH::IRF4* juxtaposition supporting the presence of a cryptic *IRF4* translocation. The failure to demonstrate an *IRF4* break with available FISH probes has been estimated to occur in approximately 10% of LBCL-*IRF4* [18, 19, 37, 43, 45]. In cryptic cases, the presence of IGH/IGK/IGL rearrangement helps to support the diagnosis. Furthermore, case LYWS-1279 had, in addition, an *IRF4* mutation. The presence of one or multiple mutations affecting the *IRF4* gene with an aberrant somatic hypermutation pattern is a hallmark of the *IRF4* translocation [37, 39, 40]. Therefore, the presence of *IRF4* mutations in the highly conserved N-terminal DNA-binding domain in exon 2, in the correct context, might be used as a surrogate marker for the presence of *IRF4* rearrangement [39, 40]. Mutational analysis performed by the panel demonstrated the presence of multiple *IRF4* mutations in 4 of 5 cases analyzed (80%) (Supplemental Table 4). The only case without *IRF4* mutations was submitted by Eric Hsi (LYWS-1202). This case is unique in that the *IRF4* rearrangement was with an unknown partner on chromosome 21, demonstrated by RNA sequencing. In all cases reported until now, the *IRF4* translocation partner is either IGH or less frequently IGL or IGK [17, 39]. The significance of a non-IG partner is not known. Surprisingly, three cases (3/5, 60%) showed pathogenic *TP53* mutations (LYWS-1279, LYWS-1163, and LYWS-1202); nevertheless, the presence of *TP53* mutation did not seem to convey a bad prognosis, since all three cases were in CR 18, 27, and 38 months after diagnosis. In one of the first studies of LBCL-*IRF4*, *TP53* mutations were identified in 3 of 6 cases with 17p loss [46]. A subsequent study in pediatric population demonstrated 17p/*TP53* deletions in 25% of the cases; however, no *TP53* mutations were identified [39]. Similar findings were reported in an adult cohort [40]. The incidence of *TP53* mutations in this disease is unknown and its prognostic significance remains to be established. FISH analyses for *BCL2*, *BCL6*, and *MYC* were performed by the panel in all cases without evidence of translocations. The four cases investigated with GEP revealed a GCB-type signature.

**Table 2:** Comparison of clinicopathological features of 22 cases with *IRF4* rearrangements

Clinicopathological features	LBCL-*IRF4* in patients < 25 years (6 cases)	LBCL-*IRF4* in patients > 25 years (9 cases)	Aggressive lymphomas with *IRF4* (7 cases)
Age	Median 8 years (range 6–25 years)	Median 69 years (range 37–87 years)	Median 75 years (range 14–92 years)
Sex	M:F 2.5:1	M:F 3.5:1	M:F 2.5:1
Localization			
-Tonsils	4 cases (57%)	2 cases (22%)	3 cases (43%)
-Lymph nodes	1 case (14%)	5 cases (56%)	2 cases (29%)
-Intestine	1 case (14%)	–	1 case (14%) soft
-others	1 case, (14%) spleen	1 case (11%) soft tissue 1 case (11%) oral lesion*	tissue 1 case (14%) testis
Clinical stage			
- Stage 1/2	7 cases (100%)	5 cases (56%)	2 cases (29%)
- Stage 3/4	0	4 cases (44%)	5 cases (71%)
Growth pattern			
-Follicular	2/6 case (33%)	3 cases (33%)	1 case (14%) (FL3A)
-Follicular/diffuse	2/6 cases (33%)	0	0
-Diffuse	2/6 cases (33%)	6 cases (67%)	6 cases (86%)
Immunophenotype			
-MUM1	7 cases (100%)	9 cases (100%)	7 cases (100%)
-CD10	6 cases (86%)	7 cases (78%)	6 cases (86%)
-BCL6	7cases (100%)	9 cases (100%)	6 cases (86%)
-BCL2	6 cases (86%)	2 cases (22%)	5 cases (71%)
-CD5	3 cases (43%)	0 cases (0%)	1 case (14%)
FISH BAP			
-*IRF4*	5/6 (83%)^§^	9 cases (100%)	7 cases (100%)
*-MYC*	0	0	2 cases (29%)
*-BCL2*	0	0	4 cases (57%)
*-BCL6*	0	1 case (11%)	0
-*CCND1*	0	0	1 case (14%)
Mutational profile			
-*IRF4*	4/5 cases^#^ (80%)	4/5 cases (80%)	1/4 case (25%)**
-*TP53*	3/5 cases	0	0
GEP			
-GCB	4/4 cases (100%)	4/4 cases (100%)	4 cases (57%)
-ABC	0	0	3 cases (43%)

*LBCL-IRF4* large B-cell lymphoma with *IRF4* rearrangement, *BAP* break apart probe, *FISH* fluorescence in situ hybridization, *GEP* gene expression profile, *GCB* germinal center B-cell, *ABC* activated B-cell, *FL3A* follicular lymphoma grade 3A

*HIV+ patient

^§^The case without *IRF4* break was a cryptic translocation demonstrated by RNAseq

^#^The case without *IRF4* mutation was the case of *IRF4* translocation with an unknown partner in chr.21

**These cases have other mutations typical of GCB and ABC diffuse large B-cell lymphomas

Case LYWS-1276 submitted by Francisco Llamas-Gutierrez was an interesting case of a 14-year-old boy who presented with a right tonsillar tumor. Although the morphology and phenotype (CD10+, BCL6+ MUM1+) were suggestive of the diagnosis of LBCL-*IRF4*, no *IRF4* break was identified by FISH, and RNAseq analysis also failed to prove the presence of an *IRF4* rearrangement. Instead, a *BCL6* translocation was demonstrated. Mutational analysis revealed typical mutations of DLBCL without *IRF4* mutations, and GEP showed an ABC-type signature. The panel agreed with the diagnosis of DLBCL, NOS, ABC-type with *BCL6* translocation, mimicking a LBCL-*IRF4*. This case highlights the importance of FISH and molecular analysis to render the correct diagnosis.

#### LBCL-IRF4 in patients >25 years

**Fig. 6:**
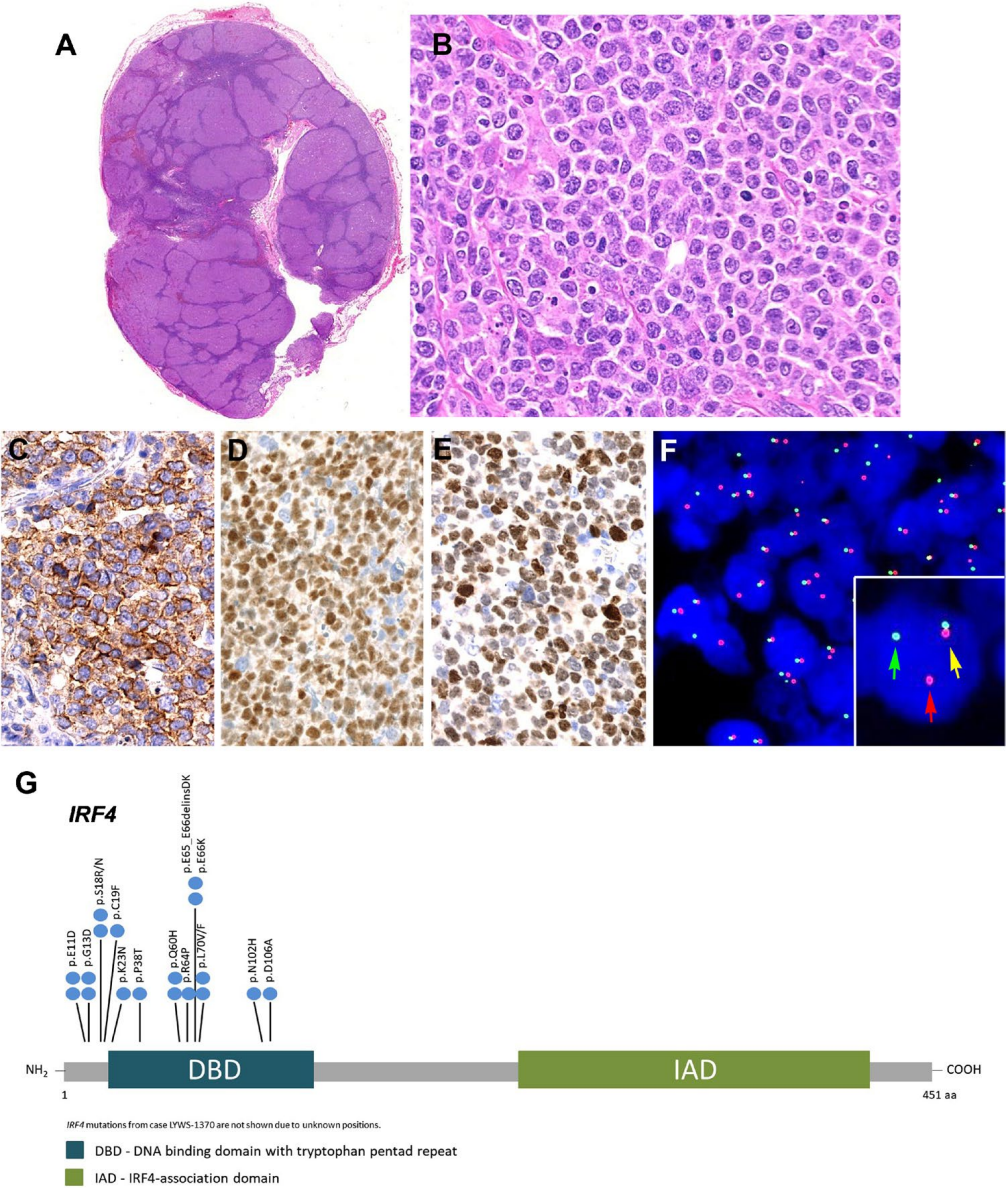
Histologic and immunophenotypic features of large B-cell lymphoma with *IRF4***-**rearrangement in an adult patient. Case LYWS-1370 courtesy of B. Bisig. **A** Panoramic view of a lymph node with preserved capsule and effacement of the architecture by the presence of multiple, large confluent nodules. No diffuse areas or necrosis are observed. **B** Higher magnification demonstrates that the nodules are composed of large cells with fine chromatin, multiple nucleolei, abundant cytoplasm typical of centroblasts. Some apoptotic figures and some mitosis are observed. **C** The tumor cells were CD10 positive as well as **D** BCL6 and **E** IRF4/MUM1. **F** Interphase FISH analysis using break apart probes for *IRF4* revealed 1 fusion signal (yellow arrow), one red signal (red arrow), and one green signal (green arrow) indicative of an *IRF4* rearrangement. **G** Diagram of the relative positions of *IRF4* driver mutations. The approximate location of somatic mutations identified is indicated. The analysis was performed by next generation sequencing. *IRF4* mutations are mainly in the DNA binding domain (DBD). Domains of the protein are represented according to the Uniprot database (www.uniprot.org)

Nine cases were submitted with this diagnosis in adult patients (Table 2 and Supplemental Table 4). There were 7 males and 2 females with a median age of 69 years (range 37–87 years). In two cases, probably because of the age of the patients, a composite lymphoma was found; one case with lymphoplasmacytic lymphoma (LPL) (LYWS-1076), and one case with a marginal zone lymphoma (LYWS-1320). In contrast to the pediatric cases, adult cases were more often nodal (5 cases) with only two tonsillar presentation, one case with forearm soft tissue involvement, and one oral lesion in an HIV+ patient. Interestingly, a recent study suggests that involvement of skin/soft tissue is relatively frequent in elderly patients, a presentation previously unrecognized [43]. Although cases in adults presented more often with high clinical stages (stages III/IV) (4/9; 44%), all patients achieved CR with R-CHOP (rituximab-cyclophosphamide, hydroxydaunorubicin, oncovine, and prednisone). Morphologically, 3 cases showed FL3B-like morphology, and 6 cases were diffuse. The immunophenotype was similar to the pediatric population (CD10+, BCL6+, BCL2+); however, CD5 expression was not demonstrated in adult patients and in two cases CD10 was negative. Case LYWS-1076 was presented by Dominik Nann and corresponded to a 74-year-old patient in clinical stage III with FL3B-like morphology, and bone marrow (BM) infiltration by a clonally unrelated LPL. This case, in addition to an *IRF4* translocation, also carried a *BCL6* translocation. *BCL6* translocations were reported in 35% (8/23) of the cases in the original description of the disease, six of them in adult patients [17]. However, out of all of the cases submitted to the workshop, both children and adults, only this case carried a *BCL6* translocation (1/14; 7%). In general, in adult patients with morphology of FL3B, as demonstrated also by case LYWS-1370, submitted by Bettina Bisig (Fig. 6A–F), it is recommended to stain for MUM1/IRF4, and if positive, to perform FISH analysis for *IRF4* [25, 47]. These cases, in contrast to FL3B cases, have an excellent prognosis as demonstrated by the cases submitted to the workshop with complete remission up to 10 years. The two cases that presented in the tonsil corresponded to a 57-year-old man (LYWS-1142, submitted by Austin Gray) and a 79-year-old woman (LYWS-1238, submitted by Konnie Hebeda), with clinical stage I or stage II, respectively. These two cases were clinically, morphologically, and genetically indistinguishable from the cases in the pediatric population confirming the existence of LBCL-*IRF4* in elderly patients indistinguishable from the pediatric counterpart [40, 43, 47]. It may also indicate that cases arising in tonsils, Waldeyer’s ring or bowel may represent a distinct clinicopathologic entity regardless of the age of the patient at disease presentation.

Of the 16 cases submitted with the diagnosis of LBCL-*IRF4*, the panel performed mutational analysis (Ion GeneStudio S5, ThermoFisher Scientific) and GEP (HTG EdgeSeq System, HTG Molecular Diagnostics) in those cases where material was available for molecular studies. Multiple *IRF4* mutations in exon 2 were demonstrated in eight cases (8/10; 80%) (Fig. 6G, Supplemental Table 6), whereas GEP demonstrated a GCB-type signature in these cases (8/8; 100%).

### Aggressive B-cell lymphomas with *IRF4 and BCL2/MYC/CCND1* rearrangements

Seven cases with *IRF4*-R associated with other genetic alterations, namely *BCL2-R*, *MYC-R*, and *CCND1-R*, were submitted to the workshop (Table 2 and Supplemental Table 5). There were 5 males and 2 females with a median age of 75 years (range 14–92). The only pediatric patient was a 14-year-old girl with a plasmablastic lymphoma (PBL) with *IGK::IRF4* and *IGH::MYC* rearrangements. The other 6 cases were elderly patients. Case LYWS-1024 was presented by Holly Berg and corresponded to a 92-year-old woman who presented with a scalp mass in stage IIIA. The case was CD10+, BCL6+, MUM1+, BCL2+, and CD5 weak positive. FISH analyses demonstrated *IRF4* and *BCL2* rearrangements. Mutational analysis revealed gene mutations characteristic of DLBCL including *KMT2D*, *CREBBP*, *BCL2*, *CCND3*, *NOTCH2*, and *DNMT3A*. No *IRF4* mutations were identified. A similar case was submitted by James Cook (case LYWS-1086). Recent studies have suggested that large B-cell lymphomas with *IRF4* and *BCL2* rearrangements in elderly patients show mutational profiles closer to DLBCL [40, 43]. Therefore, cases with both *IRF4* and *BCL2* rearrangements should be diagnosed as DLBCL, NOS. Case LYWS-1408 submitted by Maria Rodriguez-Pinilla is a unique case of 75-year-old man, who presented in stage I disease with tonsillar involvement and is in complete remission after 2 years only with rituximab. The immunophenotype was CD10+, BCL6+, MUM1+, BCL2+, cyclin D1+, and SOX11 negative. This case harbored an *IRF4*-R together with a *CCND1*-R. The panel performed mutational analysis and identified three *CD70* mutations, and *SOCS1* and *TMSB4X* mutations. Clinically, the case behaved indolently, unlike mantle cell lymphoma. The panel rendered the diagnosis of DLBCL, NOS with *IRF4* and *CCND1* rearrangements. There were two cases of FL (Cases LYWS-1099 and LYWS-1133), one grade 3A with *BCL2* and *IRF4* rearrangements, and one transformed FL to high-grade lymphoma that in the transformation acquired *MYC* and *IRF4* rearrangements. The meaning of *IRF4* rearrangements in other aggressive lymphomas is not well understood and warrants further studies. Importantly, these cases should not be diagnosed as LBCL-*IRF4.*

### Other molecular groups in large B-cell lymphomas

**Fig. 7:**
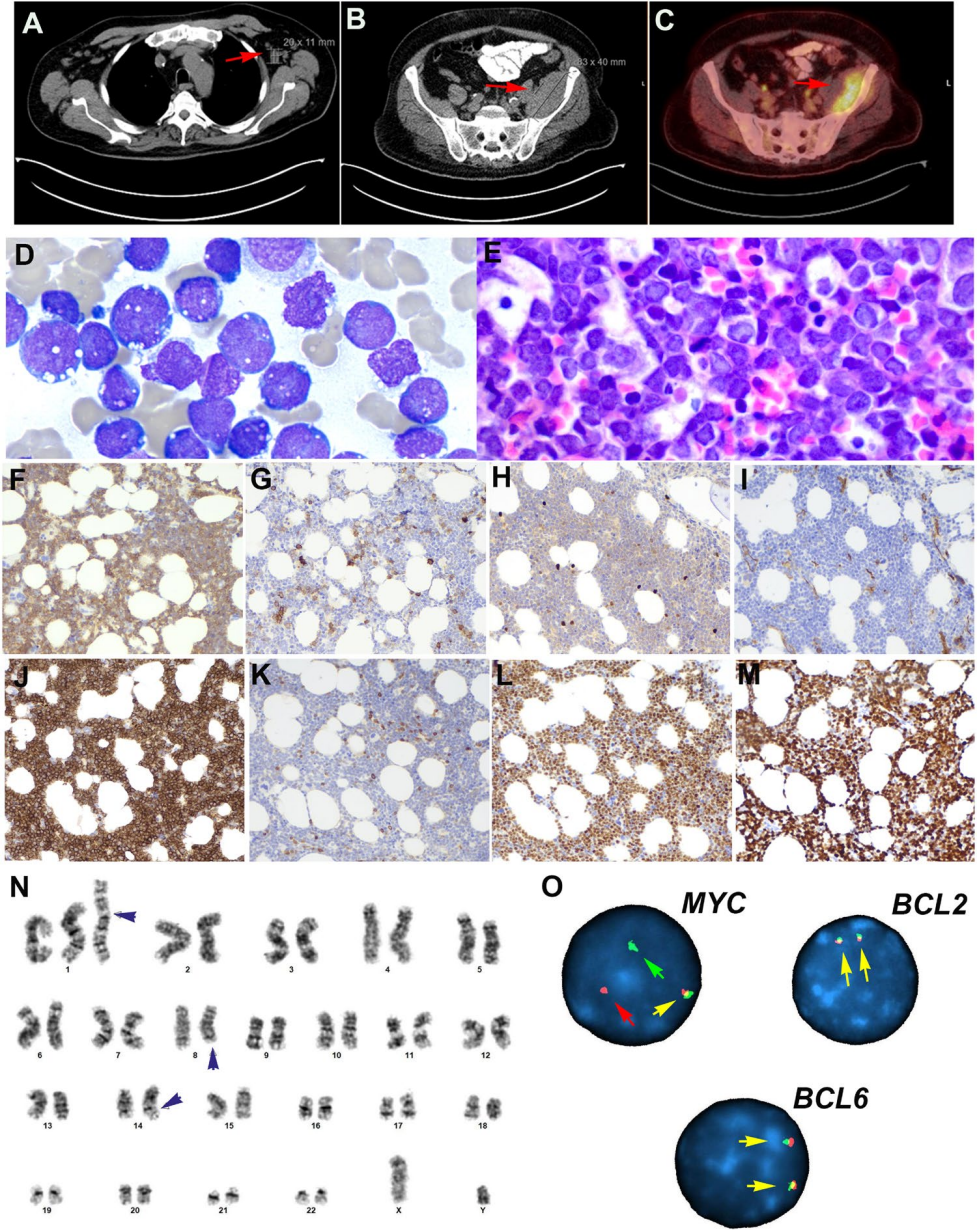
Histologic, immunophenotype, and cytogenetic features of a B-cell acute lymphoblastic leukemia with *IGH::MYC* according to the 2022 ICC. Case LYWS-1026 courtesy of G. Caponetti. **A** CT scan demonstrates supradiaphragmatic lymphadenopathy (red arrow). **B** CT scan reveals a soft tissue lesion expanding the posterior aspect of the left iliacus muscle (red arrow) **C** PET scan shows intense FDG uptake by the soft tissue mass (red arrow). **D** The bone marrow aspirate shows 81% blasts with vacuolated cytoplasm typical of Burkitt lymphoma. **E** The bone marrow biopsy is hypercellular for age and shows a diffuse infiltrate with relatively large blastoid cells. **F** The tumor cells are positive for CD19. **G** CD10, **H** TdT, and **I** CD34 are negative. **J** CD10 is strongly positive in the tumor cells. **K** BCL2 is negative, whereas **L** MYC is strongly positive. **M** The MIB1 stain shows a 100% proliferation. **N** The karyotype analysis reveals an abnormal male karyotype with a supernumerary isochromosome for the entire long arm of chromosome 1, which results in tetrasomy 1q (black arrow head). Additionally, a balanced translocation between chromosome 8 and 14 is observed with breakpoints at bands 8q24.2 and 14q32 resulting in a *IGH::MYC* translocation. **O** Interphase FISH analysis using break apart probes for *MYC*, *BCL2*, and *BCL6* reveals in *MYC* 1 fusion signal (yellow arrow), one red signal (red arrow), and one green signal (green arrow) indicative of *MYC* rearrangement. In contrast, the analyses of *BCL2* and *BCL6* demonstrate two normal fusion signals (yellow arrows)

The 13 cases included in this group were very heterogeneous and represented single examples of different molecular subgroups (Supplemental Table 7). Three cases were thoroughly discussed during the workshop representing novel concepts in lymphoma biology and classification. The case presented by Gabriel Caponetti (LYWS-1026) corresponded to a 69-year-old male who presented with 2-week history of fatigue and shortness of breath. Peripheral blood analysis demonstrated leukocytosis (57.3/L), thrombocytopenia (68,000/L) without anemia, and 28% blasts with Burkitt-like morphology (Fig. 7A–O). The patient had lymphadenopathy and splenomegaly. The BM aspirate revealed 81% blasts. Although the progenitor markers CD34 and TdT were absent, the neoplasm lacked CD20 and surface immunoglobulin (sIg) findings that can be seen with immaturity. The tumor cells expressed CD19, PAX5, CD10, MUM1, and MYC, and showed a proliferation rate of 100%. The karyotype revealed an abnormal male karyotype: 47,XY,+i(1)(q10), t(8;14)(q24.2;q32)[8]/46,XY[12]. FISH analysis confirmed the *MYC* rearrangement. Mutational analysis demonstrated a *NRAS* mutation and two *TP53* mutations. Cases similar to this have been reported in the literature as BL with supernumerary isochromosome 1q resulting in tetrasomy 1q [48–50]. However, recent studies have demonstrated that these cases are better classified as “B-acute lymphoblastic leukemia (B-ALL) with *MYC* rearrangement.” [25, 51, 52] B-ALL with *MYC* rearrangement is recognized in the 2022 ICC as a specific entity [25] and is included in the 5th WHO classification within the group of “B-ALL with other rearrangements.” [26] The disease affects mostly male patients (range 3–75 years), and the tumor cells have an immature phenotype, often with expression of TdT and CD10 without CD20, BCL6 and sIg; however lack CD34. The lack of TdT expression, as in this case, does not preclude this diagnosis. The *IG::MYC* translocation derives from an aberrant (out-of-frame) VDJ recombination in an immature B-cell, and not in a germinal center B-cell like BL [53]. Molecularly, these cases are also distinct from BL and show frequent gains in chromosome 1q21.1-q44 and mutations in *NRAS* and *KRAS* [51, 54]. The prognosis is poor, as demonstrated in this case; the patient died 5 months after the initial diagnosis. The panel agreed with the final diagnosis of B-ALL with *MYC* rearrangement.

**Fig. 8:**
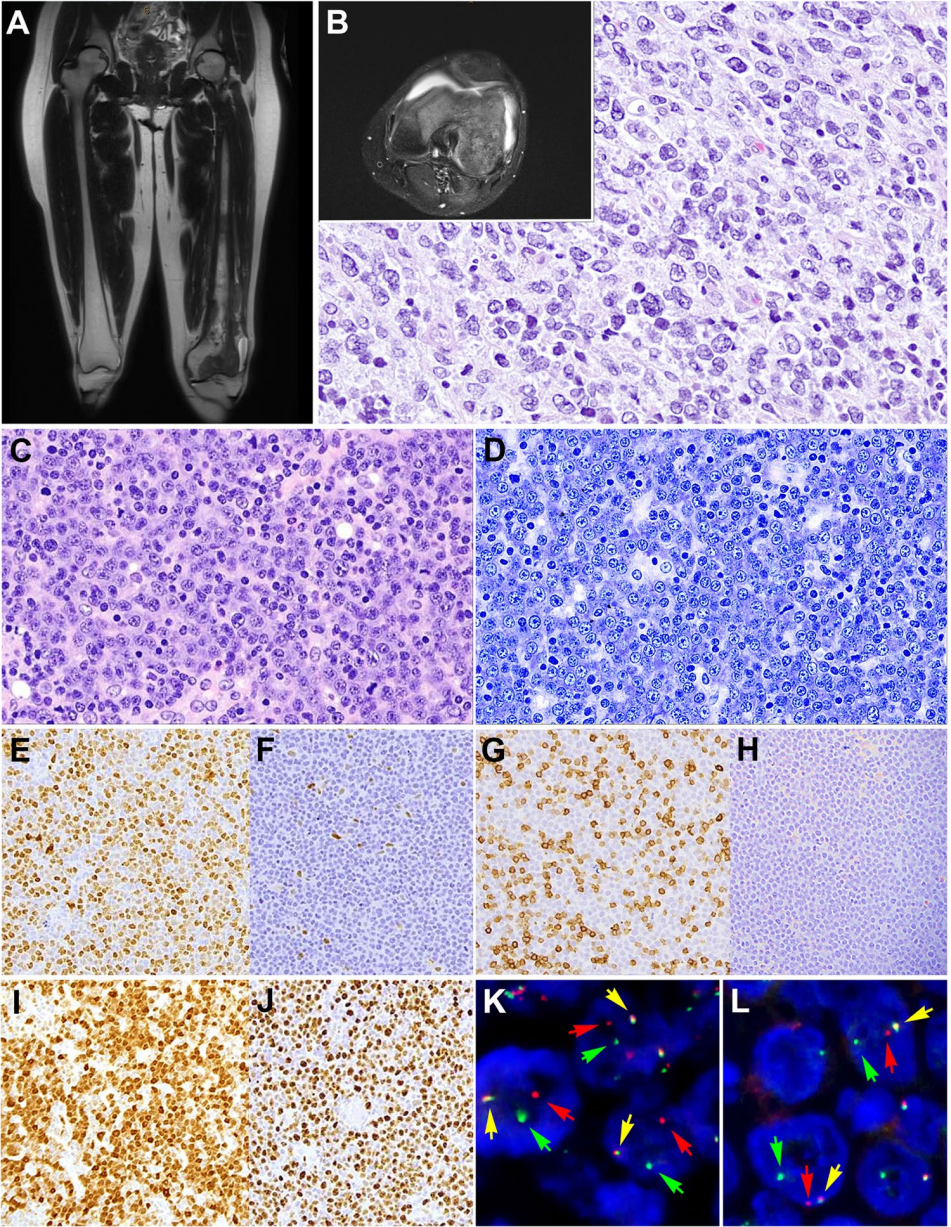
Primary diffuse large B-cell lymphoma of the bone (**A**–**B)**. Case LYWS-1231 courtesy of Y-Ch. Liu. **A**–**B** Imaging studies (MRI) of the left femur shows a lobulated mass extended from the mid diaphysis into the distal epiphysis including both the medial and lateral femoral condyles. **B** The bone biopsy showed a polymorphic infiltrate predominantly of large cells confirming the diagnosis of diffuse large B-cell lymphoma. **C**–**L** Diffuse large B-cell lymphoma with CCND1 rearrangement. Case LYWS-1380 courtesy of K.S. Kurz. **C** Lymph node biopsy with effaced architecture by a diffuse infiltrate of predominantly medium-sized to large blast cells. **D** Giemsa stain reveals that the tumor cells have basophilic cytoplasm and round to oval nuclei with open chromatin and prominent nucleolus. **E** Cyclin D1 is positive in the tumor cells. **F** In other areas of the lymph node the tumor cells were cyclin D1 negative. **G** CD5 stain is positive in the reactive T cells but negative in the tumor cells. **H** SOX11 remains negative. **I** MUM1 is positive in the majority of tumor cells whereas CD10 remained negative (not shown). **J** Ki-67 demonstrate a high proliferation rate. **K**–**L** Interphase FISH analysis using break apart probes for *CCND1* and *MYC* reveals 1 fusion signal (yellow arrow), one red signal (red arrow), and one green signal (green arrow) indicative of *CCND1* (K) and *MYC* (L) rearrangements

Another interesting case (LYWS-1231) presented by Yen-Chun Liu corresponded to a 24-year-old female who presented with left knee pain for 3 years (Fig. 8). Imaging studies demonstrated in the left femur a lobulated mass extended from the mid-diaphysis into the distal epiphysis including both the medial and lateral femoral condyles (Fig. 8A–B). No additional lesions were demonstrated. The bone biopsy was diagnostic of a DLBCL with CD20 and BCL6 positivity but negative for CD10, MUM1, cyclin D1, and EBER. Whole-genome sequence demonstrated the presence of the following mutations: *EZH2*^Y646N^, *IRF8*^Y23H^, *TNFRSF14*, and *UBR5*, and complex chromosomal alterations. The patient was treated with 2 cycles of chemotherapy (cytarabine and methotrexate) and has been in complete remission for the last 3 years. PB-DLBCL is a disease with characteristic clinical presentation, and morphological and genetic features [55, 56]. It tends to affect younger patients; occurs more frequently in the femur, followed by the pelvis, vertebrae, and humerus; and has an excellent prognosis. Unlike most extranodal lymphomas, PB-DLBCL shows a centrocyte-like GCB-type GEP and a characteristic mutational profile similar to FL including alterations in *B2M*, *EZH2*, *IRF8*, and *TNFRSF14* [55]. The distinctive clinicopathological features suggest that PB-DLBCL is an extranodal manifestation of DLBCL; however, the possibility of an extranodal t(14;18)-negative FL with predominantly large centrocytes, similar to primary cutaneous follicle center lymphoma, cannot be ruled out. Further studies are needed to characterize better this type of lymphomas.

Overexpression of cyclin D1 in DLBCL has been associated mostly with gains of *CCND1* gene copies [57, 58]. The demonstration of *IGH::CCND1* rearrangement leading to cyclin D1 overexpression can occur in DLBCL and poses a diagnostic challenge with blastoid or pleomorphic mantle cell lymphoma [59–61]. Most cases have been reported associated with other chromosomal translocations including *BCL6*, *BCL2*, and *MYC*. The translocation has been reported mostly as secondary genetic event in the evolution of DLBCL and other lymphomas such as CLL [62] and FL [63]. Accordingly, the case LYWS-1380 presented by Katrin S. Kurz corresponded to a 58-year-old man, who presented in clinical stage IIA. Morphologically, the lymph node showed a diffuse infiltration by large cells that were CD20+, MUM1+, and BCL2+ (Fig. 8C–L). The tumor cells were negative for CD10, BCL6, CD5, and SOX11. The proliferation rate was 90% and MYC was positive in around 50% of the cells. Interestingly, cyclin D1 was strongly positive only in a part of the lymph node and the rest remained cyclin D1 negative. FISH analysis with BAP for *CCND1* demonstrated a break only in those areas where cyclin D1 was positive indicating that the translocation was indeed a secondary genetic event in the evolution of this lymphoma. Moreover, *MYC-R* was also identified in the same area. The FISH analysis for *BCL2* and *BCL6* showed normal signals. Mutational analyses demonstrated several mutations (*CARD11*, *CD79B*, *FOXO1*, *PIM1*, *SOCS1*), supporting the diagnosis of DLBCL. The GEP performed by the panel confirmed the ABC-type signature. In general, cases with large cell morphology and cyclin D1 positivity but lack CD5 and SOX11 expression should raise the possibility of DLBCL over MCL. The expression of MUM1 or FOXP1 may be useful to achieve the correct diagnosis [59].

Histologic transformation of small B-cell NHL to aggressive lymphoma is well recognized. However, transformation to an aggressive lymphoma with plasmablastic morphology and phenotype is rare [64]. Plasmablastic transformation has been reported in CLL, FL, and rarely LPL [64] [65]. Although these transformed tumors mimic primary PBL, they are not associated with immunodeficiency, and rarely have EBV infection or *MYC* alterations [64]. Furthermore, they display features indicating clonal evolution from a small B-cell lymphoma and not de novo PBL. Two cases submitted to the workshop (LYWS-1167 submitted by Silvia Tse Bunding, and LYWS-1405 submitted by Anu Peter) represented nice examples of cases that progressed/transformed to an aggressive lymphoma with PBL-like features. Case LYWS-1405 was a 68-year-old man with 8 years history of CLL, who received different treatments including ibrutinib in the last 3 years. He presented with a right groin mass that was biopsied and diagnosed as PBL with a *MYC*-R. Molecular studies demonstrated that the CLL and PBL were clonally related and shared the same *TP53* mutation. Interestingly, recent reports have associated treatment of CLL with the BTK inhibitor ibrutinib and Richter’s transformation mimicking PBL [66–68]. Further studies are warranted to understand PBL-like transformation in CLL and in other lymphomas.

## Conclusions

Because of new and improved techniques used for standard diagnosis and translational research, we have witnessed a growing list of genetic alterations, variably present in large B-cell lymphomas that are useful for diagnosis or to understand the biology and pathogenesis of certain diseases. Some of these alterations (*IRF4*-R and 11q aberration) became the defining genetic alteration of new disease entities (Box 1).

HG/LBCL-11q is defined by chromosome 11q-gains and telomeric loss and can occur in the setting of immunodeficiency including posttransplant and HIV+ patients but also in patients with A-T. The disease predominates in children and has a broad morphological spectrum and a Burkitt-like immunophenotype, but MYC expression is weak or negative, lacks *MYC*-R, and harbors a different mutational profile compared to BL. LMO2 is a useful marker but expressed only in 50% the cases. FISH analysis is recommended for the diagnosis, and in ambiguous cases, other more sophisticated chromosomal analysis is warranted. Whether concurrent *TP53* mutations identified in adult patients represent clonal evolution or DLBCL with *TP53* and 11q aberrations warrants further studies. Nonetheless, the presence of *TP53* mutations conveyed a dismal prognosis in this cohort.

LBCL-*IRF4* is mainly a disease of children and young adults but it also occurs in adults and elderly patients. In contrast to children and young adults, where the disease presents more often in the Waldeyer’s ring and in clinical stages I/II, in adults presents more often as nodal disease and in more advanced clinical stages. Nevertheless, the prognosis is excellent. Morphologically and genetically, the disease seems to be the same in children and adults. The aberrant expression of CD10, BCL6 and MUM1/IRF4 should prompt to perform *IRF4* FISH analysis. In the cases submitted to the workshop, *BCL6*-R were rare when compared to other published series (7% vs 35%). In cases with FL3B, MUM1 should be performed, and if positive, *IRF4* FISH is recommended. Cryptic *IRF4*-R by FISH occur in around 10% of the cases; however, in the correct context, breaks in IGH, IGK and IGL, as well as *IRF4* mutations, support the diagnosis. The cases submitted to the workshop also raised important questions that need to be resolved in the future: (1) What is the prognostic significance of *TP53* mutations? (2) Does localized disease with a follicular growth pattern needs systemic treatment, especially in children and young adults where a watch and wait strategy seems to be justified? (3) Do cases that have been reported with ABC-type GEP belong to the same disease?

*IRF4*-R can be observed in a variety of aggressive B-cell lymphomas in association with other chromosomal translocations. The *IRF4*-R might be the initial event but also a secondary event or acquired during transformation. These cases should not be diagnosed as LBCL-*IRF4*. More studies are warranted to characterize better these cases.

Finally, novel molecular groups were also discussed highlighting challenging features with some groups needing special attention such as PB-DLBCL, B-ALL with *IGH::MYC* rearrangement, DLBCL with *CCND1*-R, and PBL-like transformation.

**Table:** 

Box 1 • HG/LBCL-11q is defined by chromosome 11q-gains and telomeric loss and can occur in the setting of immunodeficiency including post-transplant and HIV+ patients but also in patients with ataxia-telangiectasia. • FISH analysis is recommended for the diagnosis of HG/LBCL-11q. • LMO2 is a useful diagnostic marker for HG/LBCL-11q but expressed only in 50% of the cases. Burkitt lymphoma is LMO2 negative. • LBCL-*IRF4* is mainly a disease of children and young adults but it also occurs in adults and elderly patients. • LBCL-*IRF4* in children and young adults affects predominantly the Waldeyer’s ring and presents in clinical stages I/II; in contrast, in adults, it is more often a nodal disease and presents in advanced clinical stages (III/IV). • LBCL-*IRF4* is an indolent lymphoma with excellent prognosis. • Morphologically, LBCL-*IRF4* can be purely follicular, follicular/diffuse, or diffuse and often shows aberrant expression of CD10, BCL6, and MUM1. CD5 expression is not rare, especially in children. • In adults, *IRF4* rearrangements can be observed in a variety of aggressive B-cell lymphomas in association with other chromosomal alterations. These cases should not be diagnosed as LBCL-*IRF4*. • Other novel molecular groups are discussed.	

### Supplementary information

ESM 1 (DOCX 44 kb)

## Data Availability

Not applicable
