# Supplementary material for: Emerging entities: high-grade/large B-cell lymphoma with 11q aberration, large B-cell lymphoma with IRF4 rearrangement, and new molecular subgroups in large B-cell lymphomas. A report of the 2022 EA4HP/SH lymphoma workshop
Source: Virchows Arch. 2023 Aug 9;483(3):281–98. doi: 10.1007/s00428-023-03590-x (PMC10541818; doi:10.1007/s00428-023-03590-x)
Supplement: Supplementary file 1 — (DOCX 44 kb) [file 428_2023_3590_MOESM1_ESM.docx]

**Supplemental Table 1. Summary of the clinicopathological features of 20 cases submitted with the diagnosis of HG/LBCL-11q**

| **Case** | **Age** | **Sex** | **Presentation** | **Phenotype** | **FISH** | **Mutations** | **Panel diagnosis** | **Submitter** |
| --- | --- | --- | --- | --- | --- | --- | --- | --- |
| LYWS-1025 | 59 | M | Ileocecal LN | CD10+, BCL6+, LMO2+/-, MYC 20%, Ki67 95-100% | Negative for *MYC, BCL2* and *BCL6* rearrangements Gain/loss of 11q | n.a. | HG/LBCL with 11q G/L aberration | S. Dirnhofer, Basel |
| LYWS-1046 | 17 | M | Tonsillar mass | CD10+, BCL2-, BCL6+, LMO2+, MYC 10-20%, Ki67 >95% | Negative for *MYC, BCL2* and *BCL6* rearrangements Gain/loss of 11q | *NSD2, HIST1H1E, CCND3, PRDM1, MYC, TP53, FOXO1, STAT3* | HG/LBCL with 11q G/L aberration | M.A.T. Arildsen, Nashville |
| LYWS-1073 | 19 | M | 1 - Cervical LN 2 - Tonsil | CD10+, BCL2-, LMO2-, Ki67 90-100% | Negative for *MYC, BCL2* and *BCL6* rearrangements Gain/loss of 11q | *mTOR, SF3B1, TET2, MYC, PTPRD, KRAS, KDM6A* | HG/LBCL with 11q G/L aberration | K.H.R. AuYeung, Hong Kong |
| LYWS-1103 | 15 | M | Tonsillar mass | CD10+, BCL2-, BCL6+, MYC <30%, Ki67 ~100% | Negative for *MYC, BCL2* and *BCL6* rearrangements Gain/loss of 11q | Gain of ch12 *PIK3R1, GNA13, DDX3X, PHF6, RHOA* | HG/LBCL with 11q G/L aberration | S. Deshmukh-Rane Los Angeles |
| LYWS-1125 | 31 | M | n.a. | CD10+, BCL2-, BCL6+, LMO2-, MYC <5%, Ki67 >95% | Negative for *MYC, BCL2* and *BCL6* rearrangements Gain/loss of 11q | *ATM* | HG/LBCL with 11q G/L aberration | S. Mato Barcelona |
| LYWS-1129 | 13 | F | Cervical LN | CD10+, BCL2+, BCL6+, LMO2+, MYC 20-30%, Ki67 >95% | Negative for *MYC, BCL2* and *BCL6* rearrangements Gain/loss of 11q | *CREBBP, PLCG2* | HG/LBCL with 11q G/L aberration | K. Morgan New Brunswick |
| LYWS-1171 | 25 | M | Abdominal mass | CD10+, BCL2+, BCL6+, MYC <15%, Ki67 ~100% | **Amplification, no deletion of 11q** | n.a. | HGBL,NOS | F. Liu Foshan |
| LYWS-1173 | 70 | M | Abdominal mass | CD10+, BCL2-, BCL6+, LMO2-, MYC <25%, Ki67 ~100% | Negative for *MYC, BCL2* and *BCL6* rearrangements Gain/loss of 11q | n.a. | HG/LBCL with 11q G/L aberration | J. Raine London |
| LYWS-1217 | 28 | M | Neck mass Stage I BLL | CD10+, BCL2+/-, BCL6+, LMO2+, MYC 20-30%, Ki67 ~100% | Negative for *MYC, BCL2* and *BCL6* rearrangements Gain/loss of 11q | *RHOA, SESN1, MYC, NOTCH1, ATM, FOXO1* | HG/LBCL with 11q G/L aberration | L.P. De Lima Guido Miami |
| LYWS-1220 | 26 | M | Appendix Stage I large cell | CD10+, BCL2-, BCL6+, LMO2-, MYC 30%, Ki67 >95% | Negative for *MYC, BCL2* and *BCL6* rearrangements Gain/loss of 11q | *PTEN, FOXO1, ATM, GNA13* | HG/LBCL with 11q G/L aberration | K. Argyropoulos Greenwich |
| LYWS-1224 | 24 | F | Gastric ulcer Ovary High-grade morphology | CD10-, BCL2-, BCL6+, LMO2-, MYC <5%, Ki67 ~100% | Negative for *MYC, BCL2* and *BCL6* rearrangements Gain/loss of 11q | complex *TP53, KMT2D* | HG/LBCL with 11q G/L aberration | J. Fontaine Lyon |
| LYWS-1234 | 72 | F | Large tumor in colon | CD10+, BCL2-, BCL6+, LMO2-, MYC 40-50%, Ki67 ~100% | Negative for *MYC, BCL2* and *BCL6* rearrangements Gain/loss of 11q | *SETD2, HIST1H1E, PTEN, KRAS, TP53, GNA13, TCF3* | HG/LBCL with 11q G/L aberration | M. Ettrup Aalborg |
| LYWS-1237 | 7 | F | Intussusception, 4 cm lesion in colon Stage III HE bad morphology | CD10+, BCL2+, BCL6+, LMO2-, MYC >80%, Ki67 ~100% | ***MYC* rearrangement** Negative for *BCL2* and *BCL6* rearrangements Gain/loss of 11q | *ARID1A, CARD11, CCND3, CD58, CDKN2A, STAT6, TNFAIP3* | HGBL, NOS | J. Bruneau Paris |
| LYWS-1242 | 15 | M | Cervical LN Stage II Large cell | CD10+, BCL2-, BCL6+, LMO2-, MYC >40%, Ki67 ~100% | Negative for *MYC, BCL2* and *BCL6* rearrangements Gain/loss of 11q | *EP300, PRDM1* | HG/LBCL with 11q G/L aberration | S. Dotlic Zagreb |
| LYWS-1255 | 19 | M | Cervical LN Stage II | CD10+, BCL2-, BCL6+, MYC 60-70%, Ki67 >95% | Negative for *MYC, BCL2* and *BCL6* rearrangements Gain/loss of 11q | n.a. | HG/LBCL with 11q G/L aberration | B. Aqil Chicago |
| LYWS-1305 | 44 | F | Tonsil Stage IV Large cell morphology | CD10+, BCL2-, BCL6+, LMO2+, MYC >30%, Ki67>90% | Negative for MYC, BCL2 and *BCL6* rearrangements Gain/loss of 11q | *PTEN, FOXO1, VAV1* | HG/LBCL with 11q G/L aberration | L. Colomo Barcelona |
| LYWS-1345 | 20 | M | Para appendix mass Stage III | CD10+, BCL2-, BCL6+, MYC 30-40%, Ki67 >95% | Negative for *MYC, BCL2* and *BCL6* rearrangements Gain/loss of 11q | n.a. | HG/LBCL with 11q G/L aberration | V. Makarenko Boston |
| LYWS-1383 | 21 | M | Isolated cervical LN Stage I | CD10+, BCL2-, BCL6+, LMO2+, MYC >30%, Ki67 ~90% | Negative for *MYC, BCL2* and *BCL6* rearrangements Gain/loss of 11q | *RHOA* | HG/LBCL with 11q G/L aberration | J. Fontaine Lyon |
| LYWS-1442 | 15 | n.a. | Cervical LN Immunodeficiency | CD10+, BCL2-, BCL6+, MYC 40%, Ki67 ~100% | Negative for *MYC, BCL2* and *BCL6* rearrangements **Deletion of 11q** | *ATM* | HG/LBCL with 11q G/L aberration | P. Dartigues Villejuif |
| LYWS-1444 | 62 | F | Mesenterial tumor | CD10+, BCL2-, BCL6+, LMO2+, MYC 50-60%, Ki67 >95% | Negative for *MYC, BCL2* and *BCL6* rearrangements Gain/loss of 11q | *TP53, EZH2,  ATRX, PTEN, PDS5B* | HG/LBCL with 11q G/L aberration | M. Yabe New York |

LYWS: lymphoma workshop, HG/LBCL-11q: High-grade/large B-celll lymphoma with 11q aberrations; FISH: fluorescence in situ hybridization; LN: lymph node; n.a.; not available; G/L: gain/loss; F: female; M:male

**Supplemental Table 2. Mutational analyses of HG/LBCL-11q**

| **Case LYWS-1025** | | | |  | |  | |  |
| --- | --- | --- | --- | --- | --- | --- | --- | --- |
| **Gene** | **Transcript** | **Exon** | **Protein change** | **cDNA change** | | **Allele frequency** | |  |
| Not performed |  |  |  |  | |  | |  |
| **Case LYWS-1046** | | | |  | |  | |  |
| **Gene** | **Transcript** | **Exon** | **Protein change** | **cDNA change** | | **Allele frequency** | |  |
| *NSD2* | **NM_001042424.3** | 15 | p.A961T | c.2881G>A | | 35% | |  |
| *HIST1H1E* | **NM_005321.3** | 1 | p.S78R | c.234C>G | | 43% | |  |
| *PRDM1* | **NM_001198.4** | 3 | p.S220N | c.659G>A | | 46% | |  |
| *MYC* | **NM_002467.6** | 2 | pN26S | c.77A>Gl | | 46% | |  |
| *FOXO1* | **NM_002015.4** | 1 | p.T24A | c.70A>G | | 35% | |  |
| *TP53* | **ENST00000269305.4** | 2 | p.I63S | c.188T>G | | 44% | |  |
| *STAT3* | NM_00138993 | 21 | p.Y640F | c.1919A>T | | 53% | |  |
| **Case LYWS-1073** | | | |  | |  | |  |
| **Gene** | **Transcript** | **Exon** | **Protein change** | **cDNA change** | | **Allele frequency** | |  |
| *SF3B1* | NM_012433.4 | 16 | p.P780H | c.2339C>A | | 16% | |  |
| *TET2* | NM_00112720 | 3 | p.P29R | c.86C>G | | 47% | |  |
| *PTPRD* | NM_001171025.2 | 8 | p.T467N | c.1400C>A | | 14% | |  |
| *KRAS* | NM_004985.5 | 2 | p.G12C | c.34G>T | | 29% | |  |
| *KDM6A* | NM_021140.3 | 10 | p.S286C | c.857C>G | | 43% | |  |
| **Case LYWS-1103 performed by submitter** | | | |  | |  | |  |
| **Gene** | **Transcript** | **Exon** | **Protein change** | **cDNA change** | | **Allele frequency** | |  |
| *PIK3R1* | NM_002460.4 | 3 | p.N564D | c.1690A>G | | 39% | |  |
| *GNA13* | NM_006572.6 | 1 | p.M68K | c.203T>A | | 81% | |  |
| *DDX3X* | NM_001356.5 | 12 | p.V419F | c.1258G>T | | 81% | |  |
| *PHF6* | NM_001015877 | 9 | p.C280W | c.840T>G | | 81% | |  |
| *RHOA* | NM_001313947 | 2 | p.Y34F | c.101A>T | | 3% | |  |
| **Case LYWS-1125** | |  |  |  | |  | |  |
| **Gene** | **Transcript** | **Exon** | **Protein change** | **cDNA change** | | **Allele frequency** | |  |
| *ATM* | NM_000051 | 40 | p.N1983S | c.A5948G | | 93% | |  |
| **Case LYWS-1129** | |  |  |  | |  | |  |
| **Gene** | **Transcript** | **Exon** | **Protein change** | **cDNA change** | | **Allele frequency** | |  |
| *CREBBP* | NM_004380.3 | 1 | p.M1V | c.1A>G | | 32% | |  |
| *PLCG2* | NM_002661.5 | 10 | p.R268W | c.802C>T | | 47% | |  |
| **Case LYWS-1171** | | | |  | |  | |  |
| **Gene** | **Transcript** | **Exon** | **Protein change** | **cDNA change** | | **Allele frequency** | |  |
| Not  performed |  |  |  |  | |  | |  |
| **Case LYWS-1173** | | | |  | | |  |  |
| **Gene** | **Transcript** | **Exon** | **Protein change** | **cDNA change** | | **Allele frequency** | |  |
| Not  performed |  |  |  |  | |  | |  |
| **Case LYWS-1217** | | | | |  |  | | |
| **Gene** | **Transcript** | **Exon** | **Protein change** | | **cDNA change** | **Allele frequency** | | |
| *RHOA* | NM_001313947.2 | 2 | p.D45G | | c.134A>G | 11% | | |
| *SESN1* | NM_001199933 | 2 | p.L44I | | c.130C>A | 52% | | |
| *MYC* | NM_002467.6 | 2 | p.F153C | | c.458T>G | 35% | | |
| *NOTCH1* | NM_017617 | 16 | p.P832L | | c.2945C>T | 51% | | |
| *ATM* | NM_000051.4 | 37 | p.D1853V | | c.5558A>T | 51% | | |
| *FOXO1* | NM_002015.4 | 1 | p.N162Y | | c.484A>T | 33% | | |
| **Case LYWS-1220 performed by submitter** | | | | |  |  | | |
| **Gene** | **Transcript** | **Exon** | **Protein change** | | **cDNA change** | **Allele frequency** | | |
| *PTEN* | NM_000314.8 | 4 | p.A79T | | c.235G>A | 42% | | |
| *FOXO1* | NM_002015.4 | 2 | p.T24A | | c.70A>G | 19% | | |
| *ATM* | NM_000051.4 | 22 | p.P1054R | | c.3161C>G | 32% | | |
| *GNA13* | NM_001282425.1 | 4 | p.I255S | | c.764T>C | 27% | | |

| **Case LYWS-1224 performed by submitter** | | | |  |  |
| --- | --- | --- | --- | --- | --- |
| **Gene** | **Transcript** | **Exon** | **Protein change** | **cDNA change** | **Allele frequency** |
| *KMT2D* | Not reported | Not reported | Not reported | Not reported | Not reported |
| *TP53* | Not reported | Not reported | Not reported | Not reported | Not reported |

| **Case LYWS-1234** | | | |  |  |
| --- | --- | --- | --- | --- | --- |
| **Gene** | **Transcript** | **Exon** | **Protein change** | **cDNA change** | **Allele frequency** |
| *SETD2* | NM_001349370 | 11 | p.M1845T | c.5534T>C | 50% |
| *SETD2* | NM_014159.7 | 5 | p.S1530N | c.4589C>A | 20% |
| *HIST1H1E* | NM_005321 | 1 | p.A65G | c.194C>G | 18% |
| *PTEN* | NM_000314.8 | 8 | p.N323Kfs*2 | c.963dupA | 39% |
| *KRAS* | NM_033360.4 | 2 | p.G12S | c.34G>A | 16% |
| *TP53* | NM_01276760 | 2 | p.L62del | c.184_186del | 22% |
| *TP53* | NM_01126114 | 6 | p.L155del | c.633_638del | 19% |
| *GNA13* | NM_006572.6 | 1 | p.E83X | c.247G>T | 20% |
| *TCF3* | NM_001136139 | 2 | p.P29R | c.86C>G | 43% |

| **Case LYWS- 1237 performed by submitter** | | | |  |  |
| --- | --- | --- | --- | --- | --- |
| **Gene** | **Transcript** | **Exon** | **Protein change** | **cDNA change** | **Allele frequency** |
| *ARID1A* | Not reported | Not reported | Not reported | Not reported | Not reported |
| *CARD11* | Not reported | Not reported | Not reported | Not reported | Not reported |
| *CCND3* | Not reported | Not reported | Not reported | Not reported | Not reported |
| *CD58* | Not reported | Not reported | Not reported | Not reported | Not reported |
| *CDKN2A* | Not reported | Not reported | Not reported | Not reported | Not reported |
| *STAT6* | Not reported | Not reported | Not reported | Not reported | Not reported |
| *TNFAIP3* | Not reported | Not reported | Not reported | Not reported | Not reported |

| **Case LYWS- 1242** | | | |  |  |
| --- | --- | --- | --- | --- | --- |
| **Gene** | **Transcript** | **Exon** | **Protein change** | **cDNA change** | **Allele frequency** |
| *EP300* | NM_001362843 | 31 | p.M2106Cfs*28 | c.6316del | 3.7% |
| *PRDM1* | NM_001198.4 | 2 | p.Y91Afs*64 | c.43-131_70dup | 74% |

| **Case LYWS- 1255** | | | |  |  |
| --- | --- | --- | --- | --- | --- |
| **Gene** | **Transcript** | **Exon** | **Protein change** | **cDNA change** | **Allele frequency** |
| Not  performed |  |  |  |  |  |

| **Case LYWS- 1305** | | | |  |  |
| --- | --- | --- | --- | --- | --- |
| **Gene** | **Transcript** | **Exon** | **Protein change** | **cDNA change** | **Allele frequency** |
| *VAV1* | NM_001258207 | 7 | p.E243K | c.727G>A | 43% |
| *PTEN* | NM_000314 | 5 | p.F90V | c.268T>G | 50% |
| *FOXO1* | NM_002015.4 | 2 | p.N377K | c.1131T>G | 7% |

| **Case LYWS- 1345** | | | |  |  |
| --- | --- | --- | --- | --- | --- |
| **Gene** | **Transcript** | **Exon** | **Protein change** | **cDNA change** | **Allele frequency** |
| *Not*  *performed* |  |  |  |  |  |

| **Case LYWS- 1383 performed by submitter** | | | |  |  |
| --- | --- | --- | --- | --- | --- |
| **Gene** | **Transcript** | **Exon** | **Protein change** | **cDNA change** | **Allele frequency** |
| *RHOA* | Not reported | Not reported | Not reported | Not reported | Not reported |

| **Case LYWS- 1442** | | | |  |  |
| --- | --- | --- | --- | --- | --- |
| **Gene** | **Transcript** | **Exon** | **Protein change** | **cDNA change** | **Allele frequency** |
| *Not*  *performed* |  |  |  |  |  |

| **Case LYWS- 1444 performed by submitter** | | | |  |  |
| --- | --- | --- | --- | --- | --- |
| **Gene** | **Transcript** | **Exon** | **Protein change** | **cDNA change** | **Allele frequency** |
| *TP53* | Not reported | Not reported | p.S240G | Not reported | 48.5% |
| *TP53* | Not reported | Not reported | p.I251T | Not reported | 41.7% |
| *TP53* | Not reported | Not reported | p.T256K | Not reported | 42.6% |
| *ATRX* | Not reported | Not reported | p.E1466* | Not reported | 33.8% |
| *EZH2* | Not reported | Not reported | p.Y646F | Not reported | 45.3% |
| *PDS5B* | Not reported | Not reported | p.R394* | Not reported | 41.5% |
| *PTEN* | Not reported | Not reported | p.L320* | Not reported | 25% |

**Supplemental Table 3- Clinicopathological features of 7 cases of LBCL-*IRF4* in patients <25 years**

| **Case** | **Sex** | **Age** | **Presentation** | **Phenotype** | **FISH** | **Mutations** | **GEP** | **Submitter** |
| --- | --- | --- | --- | --- | --- | --- | --- | --- |
| LYWS-1279* | M | 8 | Tonsil,  Stage 1, Chx  CR 18 months | CD10+,BCL6+,MUM1+  BCL2+CD5 weak  Follicular/diffuse | *IRF4* BAP  Wild-type | *IRF4*  *TP53* | GCB | Rachel Mariani  Phoenix, USA |
| LYWS-1049 | F | 6 | Parotid mass, tonsil  Stage 2, Chx | CD10+,BCL6+,MUM1+  BCL2+/-,  Diffuse | *IRF4*-R  *BCL2, BCL6,MYC*  wild-type | *IRF4, MYD88, ARID1B, KRAS* | ND | Anna Shestakova  Salt Lake City, USA |
| LYWS-1112 | M | 7 | Ileocecal mass  Surgery only,  Stage 1 | CD10+,BCL6+,MUM1+  BCL2+/-, CD5 weak  Follicular 3B | *IRF4*-R  *BCL2, BCL6,MYC*  wild-type | ND | ND | Dehua Wang  San Diego, USA |
| LYWS-1146 | M | 25 | Tonsil,  cervical LN  Stage 2, Chx | CD10+,BCL6+,MUM1+  BCL2+  Follicular/diffuse | *IRF4*-R  *BCL2, BCL6,MYC*  wild-type | *IRF4* | GCB | Tapan Bhavsar  Washington, USA |
| LYWS-1163 | M | 18 | Spleen, stage 1  CR 12 months | CD10+,BCL6+,MUM1+  BCL2+  Follicular/diffuse | *IRF4*-R,  RNAseq IGH::*IRF4*  *BCL2, BCL6,MYC*  wild-type | *IRF4* (3 mutations)  *TP53, CARD11*,  *B2M* (2 mutations) | GCB | E. Shuyu  Tennessee, USA |
| LYWS-1202 | M | 9 | Tonsil,  cervical LN  stage 2, Chx  CR, 12 months | CD10+,BCL6+,MUM1+  BCL2+  Diffuse | *IRF4*-R partner in chr21  *BCL2, BCL6,MYC*  wild-type | *TP53, TET2, SPEN*  Duo-Seq  Chr gains 3, 8, 14, 18 | GCB | Eric His  Winston-Salem, USA |
| LYWS-1395 | F | 8 | Solitary neck lesion, lumpectomy only  Stage 1  CR 2 years | CD10+,BCL6+,MUM1+  BCL2+CD5 weak  Follicular 3B | *IRF4*::IGH  *BCL2, BCL6,MYC*  wild-type | ND | ND | Weiyang Jiang  Chongqing, China |

LYWS: lymphoma workshop; FISH; fluorescence in situ hybridization; GEP: Gene expression profile; GCB: germinal center B-cell; ND: not done

CR: complete remission, Chx: patients treated with systemic chemotherapy; *Case presented in the workshop

**Supplemental Table 4. Clinicopathological features of 9 cases of LBCL-IRF4 in patients > 25 years**

| **Case** | **Sex** | **Age** | **Presentation§** | **Phenotype** | **FISH** | **Mutations** | **GEP** | **Submitter** |
| --- | --- | --- | --- | --- | --- | --- | --- | --- |
| LYWS-1060 | M | 59 | Axillary LN  Stage 2  CR | CD10+,BCL6+,MUM1+  Diffuse | *IRF4*-R  Others ND | ND | ND | J. Balakrishna  Ohio, USA |
| LYWS-1076* | M | 74 | Submandibular  Stage 3  CR, 12 months  BM: composite LPL | CD10+,BCL6+,MUM1+  FL3B | *IRF4*-R, *BCL6*-R, IGH-R  *BCL2, MYC*  Wild-type | *IRF4* (5 mutations)  *CREBBP, BCL6* | GCB | Dominik Nann  Tübingen, Germany |
| LYWS-1139 | F | 87 | Supraclavicular  Stage 3  CR | CD10+,BCL6+,MUM1+  Diffuse | *IRF4*-R  *BCL2, BCL6, MYC*  Wild-type | ND | GCB | Syrykh  Barcelona, Spain |
| LYWS-1142 | M | 57 | Tonsil  Stage 1  CR | CD10+,BCL6+,MUM1+,BCL2+  Diffuse | *IRF4*-R  *BCL2, BCL6, MYC*  Wild-type | ND | ND | Austin Gray  Loma Linda, USA |
| LYWS-1238 | F | 79 | Tonsil  Cervical LN  Stage 2 | CD10+,BCL6+,MUM1+,BCL2+  Diffuse | *IRF4*-R  *BCL2, BCL6, MYC*  Wild-type | *IRF4* (4 mutations)  *ARID1, TET2, SMARCA4, EP300* | GCB | Konnie Hebeda  Nijmegen, Nederland |
| LYWS-1320 | M | 86 | Mesenteric LN  Composite ENMZL  CR | CD10+,BCL6+,MUM1+  FL3B | *IRF4*-R, IGH-R  *BCL6* extra copies  ENMZL: *BIRC::MALT* | ENMZL: *SPEN, ARID1B* | ND | David Hopkins  Glasgow, UK |
| LYWS-1370 | M | 69 | Submandibular  Stage 2  CR | CD10+,BCL6+,MUM1+  FL3B | *IRF4*-R  *BCL2, BCL6, MYC*  Wild-type | *IRF4* (7 mutations)  *ITPKB, HISTH1E, PIM1, MYD88, KMT2C* | GCB | Bettina Bisig  Lausanne, Swiss |
| LYWS-1275 | M | 38 | Soft tissue forearm  Stage 1E  CR | CD10+,BCL6+,MUM1+  Diffuse | *IRF4*-R  *MYC* wild type  *BCL2* and *BCL6* ND | ND | ND | Juehua Gao  Chicago |
| LYWS-1246 | M | 37 | HIV+ oral lesion  Stage IV  CR, 8 months | CD10+,BCL6+,MUM1+  Diffuse | *IRF4*-R  *BCL2, BCL6, MYC*  Wild-type | *IRF4* (5 mutations)  *CXCR4, PIM1* | ND | Gabriel Vincent  Creteil, Francia |

LYWS: lymphoma workshop; FISH; fluorescence in situ hybridization; GEP: Gene expression profile; GCB: germinal center B-cell; ND: not done

CR: complete remission, §: all patients were treated with systemic chemotherapy; BM: bone marrow; LPL: lymphoplasmacytic lymphoma

ENMZL: Extranodal marginal zone lymphoma; *Case presented in the workshop

**Supplemental Table 5. Clinicopathological features of 7 cases of aggressive lymphomas with IRF4/BCL2/BCL6/CCND1 and MYC rearrangements**

| **Case** | **Sex** | **Age** | **Presentation§** | **Phenotype** | **FISH** | **Genetics** | **Panel diagnosis** | **Submitter** |
| --- | --- | --- | --- | --- | --- | --- | --- | --- |
| LYWS-1024* | F | 92 | Scalp mass  Stage 3A  No follow-up | CD10+, BCL6+, MUM1+  BCL2+, CD5+ weak | *IRF4*-R, *BCL2*-R  *BCL6, MYC*  Wild type | *KMT2D, CREBBP, BCL2, CCND3, NOTCH2, DNMT3A* | DLBCL, NOS  GCB-type with *IRF4*-R | Holly Berg  Rochester, USA |
| LYWS-1086 | M | 72 | Tonsil  Stage 3A  No follow-up | CD10+, BCL6+, MUM1+  BCL2+, MYC+ (40%) | *IRF4*-R, *BCL2*-R, IGH-R  *BCL6, MYC*  Wild type | ND | DLBCL, NOS  GCB-type with *IRF4*-R | James Cook  Cleveland, USA |
| LYWS-1099 | M | 77 | Cervical LN  Stage 3 | CD10+, BCL6+, MUM1+  BCL2+  FL 3A | *IRF4*-R, *BCL2*-R (9%),  *BCL6, MYC*  Wild type | *TET2, DNMT3A*  (3%, CHIP) | FL 3A with *BCL2* and *IRF4* rearrangements | Ji, Yuan  Rochester, USA |
| LYWS-1133 | M | 49 | 2016 tonsil FL1/2 CR  2018 cervical LN | 2018: CD10+, BCL6+  MUM1+, BCL2+ | BCR clonal identical  2018: *IRF4*-R, *MYC*-R, *BCL2* gains. | ND | Transformed FL to HGL with *MYC* and *IRF4* rearrangements | Alberto Croci  Milano, Italy |
| LYWS-1164 | M | 78 | Testis  Stage IVB  CR after ASCT | CD10+, BCL6+, MUM1+  BCL2+ | *IRF4*-R, IGK-R  *BCL6, MYC,* IGH, IGL  Wild type | *IRF4, MYD88, CD79B, PIM1, BTG2, KMT2D*  Complex karyotype  GEP: ABC | Testicular lymphoma (C5/MCD group) | Leticia Quintanilla-Fend  Tübingen, Germany |
| LYWS-1408 | M | 75 | Tonsil  Stage 1  Treated only with rituximab,  CR 2 years | CD10+, BCL6+, MUM1+  BCL2+, cyclin D1+ | *IRF4*-R, *CCND1*-R | *CD70* (3 mutations)  *SOCS1, TMSB4X*  GEP: GCB | DLBCL, NOS  GCB-type with IRF4 and CCND1 rearrangements | Maria Rodriguez-Pinilla  Madrid, Spain |
| LYWS-1326 | F | 14 | Pelvic mass + generalized LN | CD138+, CD38+, CD20-MUM1+, CD56+, MYC+ | IGK::*IRF4*  IGH::*MYC* | ND | Plasmablastic lymphoma with *MYC* and *IRF4* rearrangements | Yunxia Ye  Chengdu, China |

LYWS: lymphoma workshop; FISH; fluorescence in situ hybridization; GEP: Gene expression profile; GCB: germinal center B-cell; ABC: Activated B-cell; ND: not done

CR: complete remission, §: all patients were treated with systemic chemotherapy; R: rearrangement; LN: lymph nodes; FL: follicular lymphoma; BCR: B cell receptor

DLBCL: diffuse large B-cell lymphoma; NOS: not otherwise specified; C5/MCD: molecular subgroup; CHIP: clonal hematopoiesis of indeterminate potential

**Supplemental Table 6. Mutational analyses of LBCL-*IRF4* cases**

| **Case LYWS-1049** | | | |  | |  | |  |
| --- | --- | --- | --- | --- | --- | --- | --- | --- |
| **Gene** | **Transcript** | **Exon** | **Protein change** | **cDNA change** | | **Allele frequency** | |  |
| *MYD88* | NM_002468 | 3 | p.S219C (atypische Variante) | c.656C>G | | 26% | |  |
| *ARID1B* | NM_001371656.1 | 2 | p.Q190* | c.568C>T | | 19% | |  |
| *IRF4* | NM_002460.4 | 2 | p.L70V | c.208C>G | | 18% | |  |
| *KRAS* | NM_033360.4 | 2 | p.I24N | c.71T>A | | 4% | |  |
| **Case LYWS-1238** | | | |  | |  | |  |
| **Gene** | **Transcript** | **Exon** | **Protein change** | **cDNA change** | | **Allele frequency** | |  |
| *IRF4* | NM_002460.4 | 3 | p.D106A | c.317A>C | | 7% | |  |
| *IRF4* | NM_002460.4 | 2 | p.K23N | c.69G>T | | 9% | |  |
| *IRF4* | NM_002460.4 | 2 | p.G13D | c.38G>A | | 23% | |  |
| *IRF4* | NM_002460.4 | 2 | p.E11D | c.33G>C | | 23% | |  |
| *ARID1A* | NM_006015.6 | 1 | p.K26Pfs*24 | c.76_79del | | 11% | |  |
| *TET2* | NM_001127208.3 | 8 | p.L1329Q | c.3986T>A | | 12% | |  |
| *SMARCA4* | NM_001128849.3 | 31 | p.L1483H | c.4448T>A | | 47% | |  |
| *EP300* | NM_001429.4 | 12 | p.I725T | c.2174T>C | | 53% | |  |
| **Case LYWS-1146** | | | |  | |  | |  |
| **Gene** | **Transcript** | **Exon** | **Protein change** | **cDNA change** | | **Allele frequency** | |  |
| *IRF4* | NM_002460.4 | 2 | p.P38T | c.112C>A | | 37% | |  |
| **Case LYWS-1163** | | | |  | |  | |  |
| **Gene** | **Transcript** | **Exon** | **Protein change** | **cDNA change** | | **Allele frequency** | |  |
| *IRF4* | NM_002460.4 | 2 | p.S18R | c.54C>G | | 4% | |  |
| *IRF4* | NM_002460.4 | 2 | p.R64P | c.191G>C | | 16% | |  |
| *IRF4* | NM_002460.4 | 2 | p.Q60H | c.180G>C | | 16% | |  |
| *B2M* | NM_004048.4 | 2 | p.C45S | c.134G>C | | 7% | |  |
| *B2M* | NM_004048.4 | 2 | p.? splice site | c.68-2A>G | | 8% | |  |
| *TP53* | NM_000546.6 | 5 | p.C141Y | c.422G>A | | 8% | |  |
| *CARD11* | NM_032415.7 | 9 | p.D401N | c.1201G>A | | 19% | |  |
| **Case LYWS-1370** | |  |  |  | |  | |  |
| **Gene** | **Transcript** | **Exon** | **Protein change** | **cDNA change** | | **Allele frequency** | |  |
| *IRF4* | NM_002460.4 | 2 | 7 mutations | aSHM | | 3-9% | |  |
| *ITPKB* | NM_002221.3 | 2 | p.Gly222Trp | c.664G>T | | 49% | |  |
| *HISTH1E* | NM_005321.2 | 1 | p.Ala199Thr | c.595G>A | | 23% | |  |
| *PIM1* | NM_001243186.1 | 4 | p.Ser237Arg | c.711C>G | | 16% | |  |
| *MYD88* | LRG_157t1 | 1 | p.Ala6Profs+39 | c.16_34del | | 12% | |  |
| *KMT2C* | NM_170606.2 | 14 | p.Tyr800Phe | c.2399A>T | | 4% | |  |
| **Case LYWS-1076** | |  |  |  | |  | |  |
| **Gene** | **Transcript** | **Exon** | **Protein change** | **cDNA change** | | **Allele frequency** | |  |
| *IRF4* | NM_002460.4 | 2 | p.L70F | c.208C>T | | 50% | |  |
| *IRF4* | NM_002460.4 | 2 | p.E66K | c.196G>A | | 34% | |  |
| *IRF4* | NM_002460.4 | 2 | p.E65_E66delinsDK | c.195_196delinsCA | | 12% | |  |
| *IRF4* | NM_002460.4 | 2 | p.E65_E66delinsDK | c.195_196delinsTA | | 3% | |  |
| *CREBBP* | NM_004380.3 | 15 | p.D1009Vfs*11 | c.134G>C | | 31% | |  |
| *CREBBP* | NM_004380.3 | 13 | p.Q786* | c.2356C>T | | 17% | |  |
| *BCL6* | NM_001706.5 | 5 | p.E227* | c.679G>T | | 15% | |  |
| *BCL6* | NM_001706.5 | 5 | p.A235G | c.704C>G | | 14% | |  |
| *MYD88* | NM_002468 | 5 | p.L265P | c.794T>C | | 5% | |  |
| **Case LYWS-1246, performed by submitter** | | | |  | |  | |  |
| **Gene** | **Transcript** | **Exon** | **Protein change** | **cDNA change** | | **Allele frequency** | |  |
| *IRF4* | NM_002460.4 | 3 | p.N102H |  | | 32% | |  |
| *IRF4* | NM_002460.4 | 2 | p.E11D |  | | 1% | |  |
| *IRF4* | NM_002460.4 | 2 | p.G13D |  | | 2% | |  |
| *IRF4* | NM_002460.4 | 2 | p.S18N |  | | 1% | |  |
| *IRF4* | NM_002460.4 | 2 | p.C19F |  | | 1% | |  |
| *CXCR4* |  |  |  |  | |  | |  |
| *PIM1* |  |  |  |  | |  | |  |
| **Case LYWS-1279, performed by submitter** | | | |  | | |  |  |
| **Gene** | **Transcript** | **Exon** | **Protein change** | **cDNA change** | | **Allele frequency** | |  |
| *IRF4* | NM_002460.4 | 2 | p.Q60H | 180G>C | |  | |  |
| *TP53* | NM_000546.6 |  | splice site | 375+2T>A | |  | |  |
| **Case LYWS-1408** | | | | |  |  | | |
| **Gene** | **Transcript** | **Exon** | **Protein change** | | **cDNA change** | **Allele frequency** | | |
| *CD70* | NM_001252.5 | Splicesite Ex.2 | p.? | | c.196+1G>T | 34% | | |
| *CD70* | NM_001252.5 | 1 | p.L26S | | c.77T>C | 47% | | |
| *CD70* | NM_001252.5 | 1 | p.A22D | | c.65C>A | 90% | | |
| *SOCS1* | NM_003745.1 | 2 | p.V2I | | c.4G>A | 47% | | |
| *TMSB4X* | NM_021109.4 | 2 | p.D3E | | c.9C>G | 90% | | |
| **Case LYWS-1320** | | | | |  |  | | |
| **Gene** | **Transcript** | **Exon** | **Protein change** | | **cDNA change** | **Allele frequency** | | |
| *ARID1B* | NM_001371656.1 | 21 | p.Q2021* | | c.6061C>T | 8% | | |
| *SPEN* | NM_015001.3 | 3 | p.Y201C | | c.602A>G | 40% | | |

**Supplemental Table 7. Summary of 13 cases in other molecular groups in DLBCL**

| Case | Submitter | Panel diagnosis | Comments |
| --- | --- | --- | --- |
| LYWS-1026* | Belen Quereda-Bernabeu and Gabriel Caponetti  Pennsylvania, USA | B-ALL with IGH::*MYC* |  |
| LYWS-1231* | Yen-Chun Liu  Memphis, USA | Primary bone DLBCL |  |
| LYWS-1380* | Katrin S Kurz  Stuttgart, Germany | DLBCL with *CCND1*-R and *MYC*-R | Secondary gene event |
| LYWS-1210 | Young-Ha Oh  South Korea | EBV+ follicular lymphoma | Submitted as EBV+ germinotropic lymphoma |
| LYWS-1109 | Andrew Feldman,  Rochester, USA | Primary mediastinal B-cell lymphoma | Triple hit after treatment |
| LYWS-1069 | Andrew Allbee,  Pennsylvania, USA | DLBCL, GCB-type | Refractory to therapy with high mutational burden due to *PMS2* loss |
| LYWS-1093 | Meredith Nicols,  Cleveland, USA | Pediatric-type follicular lymphoma |  |
| LYWS-1167 | Silvia Tse Bunting  Florida, USA | High-grade double-hit lymphoma with plasmablastic lymphoma-like progression after treatment | -loss of B-cell markers after treatment  -Genetic progression |
| LYWS-1179 | Daneshpajouhnejad,  Pennsylvania, USA | DLBCL, non-GCB type | -CD10 expression  -ABC-type mutation |
| LYWS-1239 | April Chiu  Mayo Clinic, USA | ALK+ DLBCL | -82-year-old male  -Intussusception due to a 1.2 cm mass  -Surgical resection  -Stage 1 |
| LYWS-1280 | Xiang-Nan Jiang  China | EBV+ DLBCL, NOS |  |
| LYWS-1329 | Jagmohan S.Sodhu, Johnson City, USA | Testicular DLBCL with MCD/C5 genotype | Relapsed in LN |
| LYWS-1405 | Anu Peter, Pennsylvania, USA | Richter´s transformation as plasmablastic lymphoma-like | -The patient was treated with Ibrutinib  -Clonally related, same *TP53* mutation |

LYWS: lymphoma workshop; DLBCL: diffuse large B-cell lymphoma; GCB: germinal center B-cell;

LN: lymph node; B-ALL: B acute lymphoblastic leukemia; ABC: Activated B-cell

*Cases presented during the workshop.
